# Metabolite profiling of non‐sterile rhizosphere soil

**DOI:** 10.1111/tpj.13639

**Published:** 2017-08-31

**Authors:** Pierre Pétriacq, Alex Williams, Anne Cotton, Alexander E. McFarlane, Stephen A. Rolfe, Jurriaan Ton

**Affiliations:** ^1^ Plant Production and Protection (P^3^) Institute for Translational Plant & Soil Biology Department of Animal and Plant Sciences The University of Sheffield Sheffield S10 2TN UK; ^2^ biOMICS Facility Department of Animal and Plant Sciences The University of Sheffield Sheffield S10 2TN UK; ^3^ Department of Animal and Plant Sciences The University of Sheffield Sheffield S10 2TN UK

**Keywords:** rhizosphere chemistry, rhizosphere microbiome, root exudates, metabolomics, *Arabidopsis thaliana*, maize, soil, benzoxazinoids, technical advance

## Abstract

Rhizosphere chemistry is the sum of root exudation chemicals, their breakdown products and the microbial products of soil‐derived chemicals. To date, most studies about root exudation chemistry are based on sterile cultivation systems, which limits the discovery of microbial breakdown products that act as semiochemicals and shape microbial rhizosphere communities. Here, we present a method for untargeted metabolic profiling of non‐sterile rhizosphere soil. We have developed an experimental growth system that enables the collection and analysis of rhizosphere chemicals from different plant species. High‐throughput sequencing of *16S* rRNA genes demonstrated that plants in the growth system support a microbial rhizosphere effect. To collect a range of (a)polar chemicals from the system, we developed extraction methods that do not cause detectable damage to root cells or soil‐inhabiting microbes, thus preventing contamination with cellular metabolites. Untargeted metabolite profiling by UPLC‐Q‐TOF mass spectrometry, followed by uni‐ and multivariate statistical analyses, identified a wide range of secondary metabolites that are enriched in plant‐containing soil, compared with control soil without roots. We show that the method is suitable for profiling the rhizosphere chemistry of *Zea mays* (maize) in agricultural soil, thereby demonstrating the applicability to different plant–soil combinations. Our study provides a robust method for the comprehensive metabolite profiling of non‐sterile rhizosphere soil, which represents a technical advance towards the establishment of causal relationships between the chemistry and microbial composition of the rhizosphere.

## Significance Statement

Multitrophic interactions in the rhizosphere are critical for plant growth and health, and are influenced by root exudates and their microbial breakdown products. In this study, we describe a straightforward method for metabolic profiling of non‐sterile rhizosphere soil, which represents an efficient technique to identify novel semiochemicals that shape the microbial community structure and activity of the rhizosphere.

## Introduction

Plant roots convert their associated soil into complex mesotrophic environments that support a highly diverse microbial community (Dessaux *et al*., 2016). This so‐called rhizosphere effect is mediated by the exudation of plant metabolites from roots (Badri and Vivanco, 2009; van Dam and Bouwmeester, 2016; Oburger and Schmidt, 2016). The chemical composition of these root exudates and their microbial breakdown products plays a crucial role in rhizosphere interactions between plants and beneficial soil microbes (Oburger and Schmidt, 2016). Although developments in sequencing technology have revolutionized our ability to characterize rhizosphere microbial communities (van Dam and Bouwmeester, 2016; Oburger and Schmidt, 2016), the chemical diversity of the rhizosphere remains largely unexplored. This knowledge gap mostly arises from a lack of suitable methods to collect and comprehensively analyse metabolites from non‐sterile rhizosphere soil.

It has been estimated that plants exude up to 21% of their carbon through their roots, where it is metabolized by the microbial community in the rhizosphere (Hinsinger *et al*., 2006; Badri and Vivanco, 2009; Neumann *et al*., 2009). Hence, plant roots drive multitrophic interactions in the rhizosphere via root exudation chemistry. Apart from serving as a primary carbon source for rhizosphere microbes, root exudates can influence rhizosphere interactions via selective biocidal and/or signalling activity (Berendsen *et al*., 2012). Both polar and apolar compounds have been reported to influence rhizosphere interactions. In addition to polar primary metabolites, such as organic and amino acids (Rudrappa *et al*., 2008; Ziegler *et al*., 2015; van Dam and Bouwmeester, 2016), more complex apolar secondary metabolites, like flavonoids, coumarins and benzoxazinoids (Hassan and Mathesius, 2012; Neal *et al*., 2012; Ziegler *et al*., 2015; Szoboszlay *et al*., 2016), have been reported to play an important role in influencing rhizosphere microbes. For instance, the benzoxazinoid DIMBOA, which is exuded by roots of maize seedlings, has chemotactic properties on *Pseudomonas putida* KT2440 (Neal *et al*., 2012), a rhizobacterial strain that primes host defences against herbivores (Neal and Ton, 2013). Likewise, the release of malic acid from *Arabidopsis thaliana* (Arabidopsis) roots attracts the Gram‐positive rhizobacteria *Bacillus subtilis*, which in turn induces disease resistance against *Pseudomonas syringae* pv. *tomato* (Rudrappa *et al*., 2008). Furthermore, it was shown recently that plant‐derived flavonoids have profound effects on the structure of soil bacterial communities (Szoboszlay *et al*., 2016). Although these studies illustrate the importance of specific classes of root‐derived chemicals in rhizosphere interactions, untargeted metabolome studies of root exudation products remain scarce, thereby limiting the scope for discoveries of important rhizosphere signals (Lakshmanan *et al*., 2012; Neal *et al*., 2012).

In addition to plant genotype and nutrition, various other factors can influence root exudation chemistry, such as plant developmental stage, temperature, humidity and physiochemical soil properties (Boyes *et al*., 2001; Uren, 2007; Badri and Vivanco, 2009; Zhang *et al*., 2016). The environmental effects of root exudation chemistry have been studied mostly in (semi)sterile hydroponic systems (Song *et al*., 2012; Vranova *et al*., 2013; da Silva Lima *et al*., 2014). An important justification for the use of such soil‐free growth conditions is that they allow for the tight maintenance of environmental variables (Ziegler *et al*., 2015; Bowsher *et al*., 2016). In addition, hydroponic growth systems prevent sorption of metabolites to soil particles and microbial degradation. A recent study made a compelling case for the use of sterile root systems for studying root exudation chemistry by demonstrating that root exudates collected from non‐sterile systems underestimated the quantity and diversity of carbon‐containing metabolites resulting from microbial breakdown (Kuijken *et al*., 2014). Using hydroponically grown roots under sterile conditions, Strehmel *et al*. (2014) reported wide‐ranging chemical diversity in root exudates of Arabidopsis, including mostly secondary metabolites such as (deoxy)nucleosides, anabolites and catabolites of glucosinolates, derivatives of phytohormones (e.g. salicylic acid, SA; jasmonic acid, JA; and oxylipins) and phenylpropanoids (e.g. coumarins and hydroxynammic acids). Nonetheless, there are disadvantages to hydroponically grown, sterile root systems. Hydroponically cultivated roots often develop root morphologies that differ from those of soil‐grown roots, which probably reflects an underlying difference in physiology that may affect exudation chemistry (Sgherri *et al*., 2010; Tavakkoli *et al*., 2010). Furthermore, microbial degradation products of root exudates, rather than the root‐exuded plant metabolites themselves, might act as potent rhizosphere signals. For instance, benzoxazinoids exuded from cereal roots can be converted into stable 2‐aminophenoxazin‐3‐one, which has strong antimicrobial and allelopathic activities (Atwal *et al*., 1992; Macías *et al*., 2005). In addition, it is plausible that certain root exudation products stimulate the production of signalling and/or biocidal compounds by rhizosphere microbes (Cameron *et al*., 2013). Therefore, ignoring the rhizosphere microbiome by studying sterile root systems limits the identification of novel semiochemicals that can shape microbial communities and their activities in the rhizosphere (Prithiviraj *et al*., 2007).

To date, various methods have been described to collect root exudates from non‐sterile rhizosphere soil. These methods have been used mostly to determine total organic carbon and/or nitrogen content (Phillips *et al*., 2008; Yin *et al*., 2014), or to assay for biological response activity (Khan *et al*., 2002). Some of these studies revealed biological activities by amino acids, organic acids and other extractable elements (Haase *et al*., 2008; Chaignon *et al*., 2009; Bravin *et al*., 2010; Shi *et al*., 2011; Oburger *et al*., 2013); however, the lack of comprehensive metabolic analyses of non‐sterile rhizosphere soil limits our ability to establish relationships between microbial community structure and rhizosphere chemistry. Here, we describe a method for untargeted metabolite profiling from non‐sterile rhizosphere soil with high microbial diversity. We have developed methods for extraction of polar and apolar metabolites that do not cause detectable levels of damage to root cells, nor affect the viability of soil‐ and rhizosphere‐inhabiting microbes. Using UPLC‐Q‐TOF mass spectrometry followed by uni‐ and multivariate statistical analyses, we demonstrate quantitative and qualitative differences in metabolite profiles between soil without plants and soil with plants, and putatively identify the rhizosphere metabolites that are enriched in extracts from soil hosting Arabidopsis and *Zea mays* (maize). We discuss the potential of this technique for discovering semiochemicals that shape microbial community structure and activity in the rhizosphere.

## Results

### Development of a plant cultivation system for the extraction of rhizosphere chemicals

**Figure 1:**
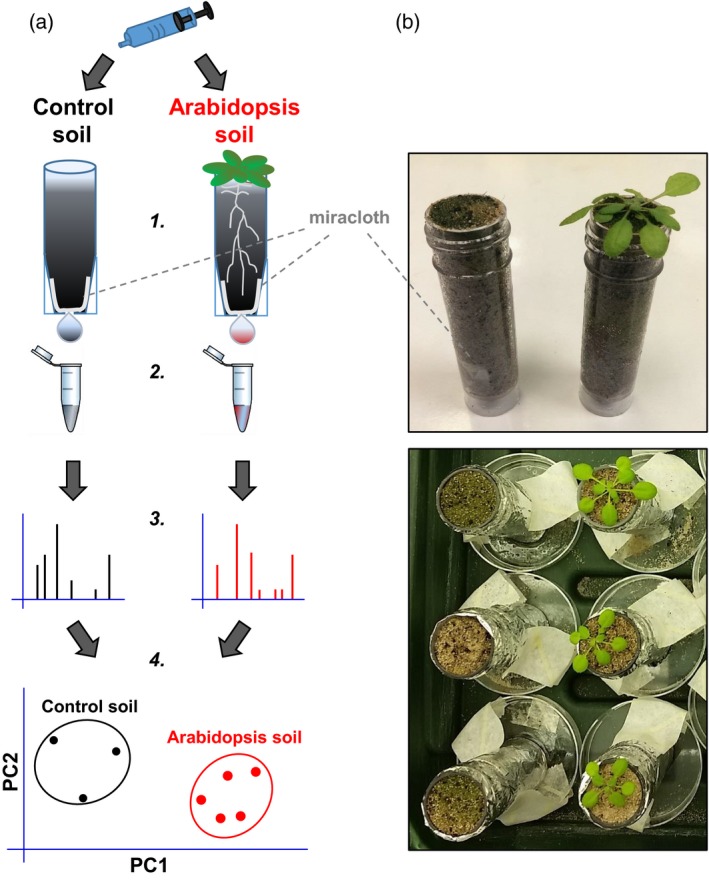
Experimental growth system and analytical approach for comprehensive chemical profiling of non‐sterile rhizosphere soil. (a) 1. Collection tubes (30 mL) with holes in the bottom (7 mm) covered by Miracloth were filled with a sand : compost mixture (9 : 1, v/v) and wrapped in aluminium foil to prevent excess algal growth. Individual Arabidopsis plants (Col‐0) were grown for 5 weeks in tubes. Additional tubes containing control soil without plants were maintained under similar conditions. 2. After the application of 5 mL of extraction solution, metabolite samples were collected for 1 min, centrifuged and freeze‐dried. 3. Concentrated samples were analysed by ultra‐high‐pressure liquid chromatography coupled with quadrupole time‐of‐flight mass spectrometry (UPLC‐Q‐TOF). 4. Multi‐ and univariate statistical methods were used to determine qualitative and quantitative differences between extracts from control soil and Arabidopsis soil. The selection of ions by statistical difference and fold‐change between soil types enabled the putative identification of metabolites that were enriched in non‐sterile rhizosphere soil. (b) Photographs of the experimental system. Top: tubes after 4.5 weeks of growth. Bottom: tubes after 3 weeks of growth taped onto Petri dishes to prevent any cross contamination of metabolites and microbes.

We used the model plant species *A. thaliana* (Arabidopsis) to develop a plant cultivation system that is suitable for the extraction of rhizosphere chemicals. Individual plants were grown for 5 weeks in 30‐ml plastic tubes with drainage holes in the bottom (Figure 1). As Arabidopsis naturally grows in sandy soils (Lev‐Yadun and Berleth, 2009), the tubes contained a homogenous 1 : 9 (v/v) mixture of fresh M3 compost and sand. Control tubes without plants were included for the extraction of chemicals from control soil. All tubes were placed in individual trays, in order to prevent any cross‐contamination of microbes and chemicals (Figure 1). Each tube was watered once per week (5 ml) from the base, with a final watering 3 days before sampling (relative water content after sampling of 88 ± 4.5% per g). This watering regime provided reproducible levels of relative water content at the time of sampling. Under these conditions, flushing the tubes with 5 ml of water or extraction solution (see below) consistently yielded 4.0–4.5 ml collected volume after 1 min of incubation.

### Microbial diversity of roots and rhizosphere soil, and rhizosphere effect

**Figure 2:**
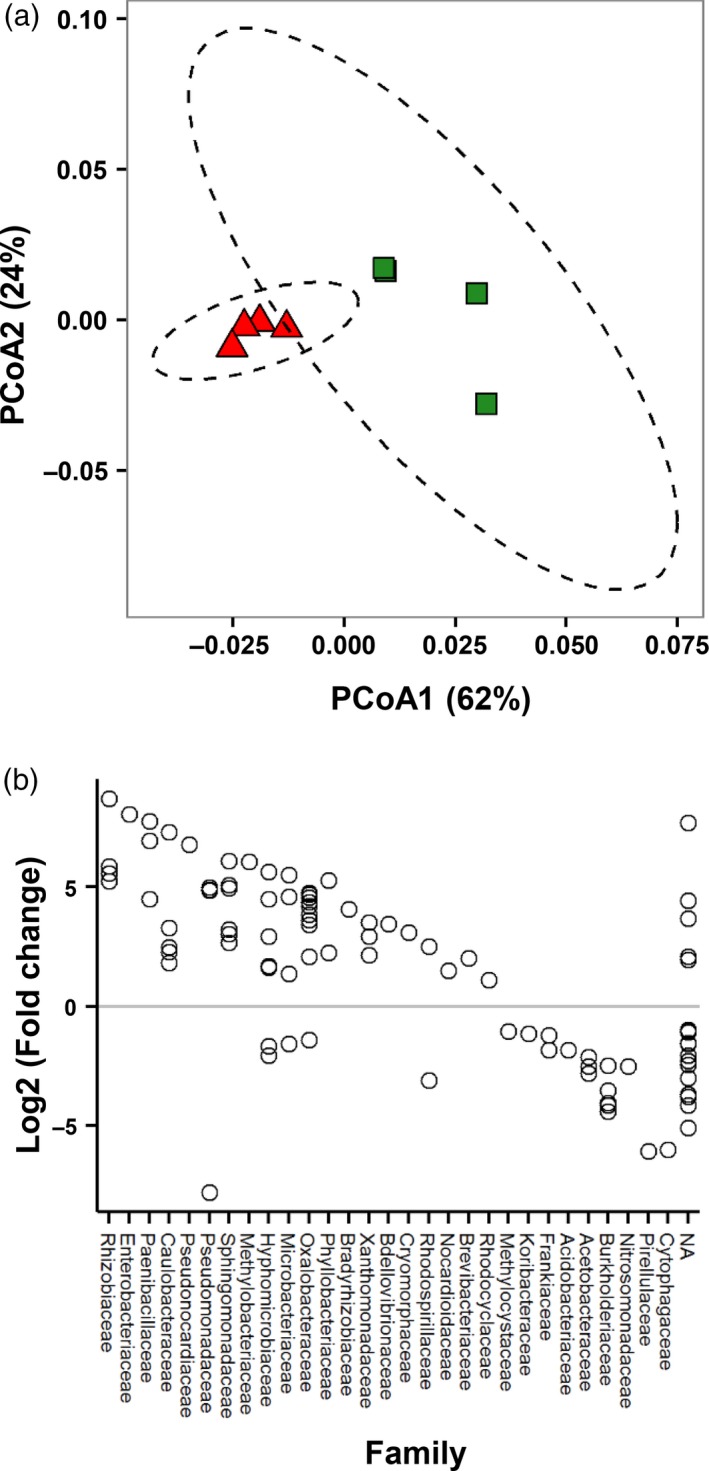
Rhizosphere effect of Arabidopsis in the cultivation system based on *16S* rRNA gene sequencing Shown are comparisons of bacterial communities between samples from control soil (without roots) and root samples plus adhering rhizosphere soil. (a) Principal coordinate analysis of operational taxonomic units (OTUs) in root plus rhizosphere samples (red) and in control soil samples (green). Ordinations were performed using weighted UniFrac distances. permanova analysis showed that the root and control soil samples differed significantly (*P* = 0.023). (b) OTUs that differ in relative abundance between root plus rhizosphere samples and control soil samples. OTUs with positive fold changes are more abundant in the root plus rhizosphere samples than in the control samples. Results are plotted by family for OTUs that showed a significant difference in abundance as calculated using DESeq2, and corrected for false discovery. Only OTUs that have a mean count of ≥20 are shown for clarity. NA, taxonomy not available.

Root‐derived chemicals mediate the rhizosphere effect (Jones *et al*., 2009; Bakker *et al*., 2013). To verify whether plants in our cultivation system showed a rhizosphere effect, we extracted DNA from control soil (without plants) and Arabidopsis roots plus adhering rhizosphere soil. Thus, the ‘root plus rhizosphere’ samples capture the microbial diversity of the rhizosphere, the rhizoplane and the root cortex. Paired‐end 250‐bp MiSeq Illumina sequencing of amplified partial *16S* rRNA genes was used to profile microbial communities. A total of 2 280 754 raw sequences were obtained with an average of 285 094 per sample. Of these, 1 693 274 reads passed quality controls, chimera removal and singleton removal. Operational taxonomic units (OTUs) were generated by clustering at 97% similarity, and were cross‐referenced against the Greengenes 13.8 database (DeSantis *et al*., 2006), yielding a total of 3863 OTUs. Rarefaction analysis (Figure S1) indicated sufficient sequencing depth to capture the majority of OTUs. Dominant bacterial taxa at the phylum level were *Actinobacteria* (10.0% across all samples) and *Proteobacteria* (87.8%), mostly comprising α*‐*, β*‐* and γ*‐proteobacteria* (17.1, 44.8 and 25.3%, respectively), whereas at the family level we detected Burkholderiaceae (16.6% across all samples), Oxalobacteraceae (16.4%), Pseudomonadaceae (14.6%) and Xanthomonadaceae (10.3%; Figure S2). In addition, we detected ten families of the Rhizobiales (9.1%), including Bradyrhizobiaceae (3.4%) and Rhizobiaceae (1.6%). Many of these phyla and families have previously been reported to be associated with plant roots (Lundberg *et al*., 2012; Bulgarelli *et al*., 2015), illustrating that the soil substrate of our cultivation system harbours a microbiome that is typical for microbe‐rich soil. To investigate whether the growth system produced a rhizosphere effect by plant roots, we analysed samples for statistically significant differences in OTUs between ‘root plus rhizosphere’ samples and soil samples. To minimize confounding effects from low‐abundance OTUs, data were filtered to include only sequences that appeared (i) more than five times across 30% of the samples, and (ii) more than 20 times across all samples, resulting in a final selection of 662 OTUs. Principal coordinate analysis (PCoA) using UniFrac distances revealed a difference in phylogenetic similarity (Figure 2a) between the ‘root plus rhizosphere’ samples and the control soil samples, which was confirmed by permanova analysis (*F*_*1*,6_, *P* = 0.023). A total of 178 OTUs were found to differ significantly in relative abundance between ‘root plus rhizosphere’ and control soil samples, including an increased abundance of 17 *Rhizobiales* OTUs in root samples (e.g. Rhizobiaceae, Methylobacteriaceae, Hyphomicrobiaceae, Phyllobacteriaceae and Bradyrhizobiaceae; Figure 2b). Although the mean Shannon diversity index did not differ between soil and ‘root plus rhizosphere’ samples (3.58, SD = 0.001; 3.22, SD = 0.001, respectively; Student's *t*‐test, *t*(3) = 0.92, *P* = 0.39), the mean OTU richness of ‘root plus rhizosphere’ samples (717, SD = 2.1) was significantly lower than that of control soil samples (1177, SD = 2.3; Student's *t*‐test, *t*(3) = 3.51, *P* = 0.04; Figure S1), showing an influence of roots on the microbial communities. Hence, the presence of plant roots in our experimental system produces a statistically significant rhizosphere effect.

### Selection of extraction solutions that do not cause detectable damage to root and microbial cells

Plant‐derived metabolites range from polar/hydrophilic (e.g. organic and amino acids, nucleotides) to apolar/hydrophobic (e.g. lipids and phenylpropanoids). Consequently, comprehensive metabolic profiling of rhizosphere soil requires extraction solutions of different polarities; however, the extraction solution should not damage cells from roots or soil microbes, which could contaminate the extract with cellular metabolites (for a conceptual model, see Figure S3). Although water‐based solutions without organic solvents are unlikely to cause cellular damage, they are unsuitable for extracting apolar (hydrophobic) metabolites. Conversely, solutions containing organic solvents extract apolar compounds, but risk cell damage by destabilization of membrane lipids (Patra *et al*., 2006). With a polarity index of 5.1, methanol (MeOH) is capable of extracting polar and apolar metabolites (Figure S3). Accordingly, we selected MeOH as the organic solvent in our extraction solutions.

**Figure 3:**
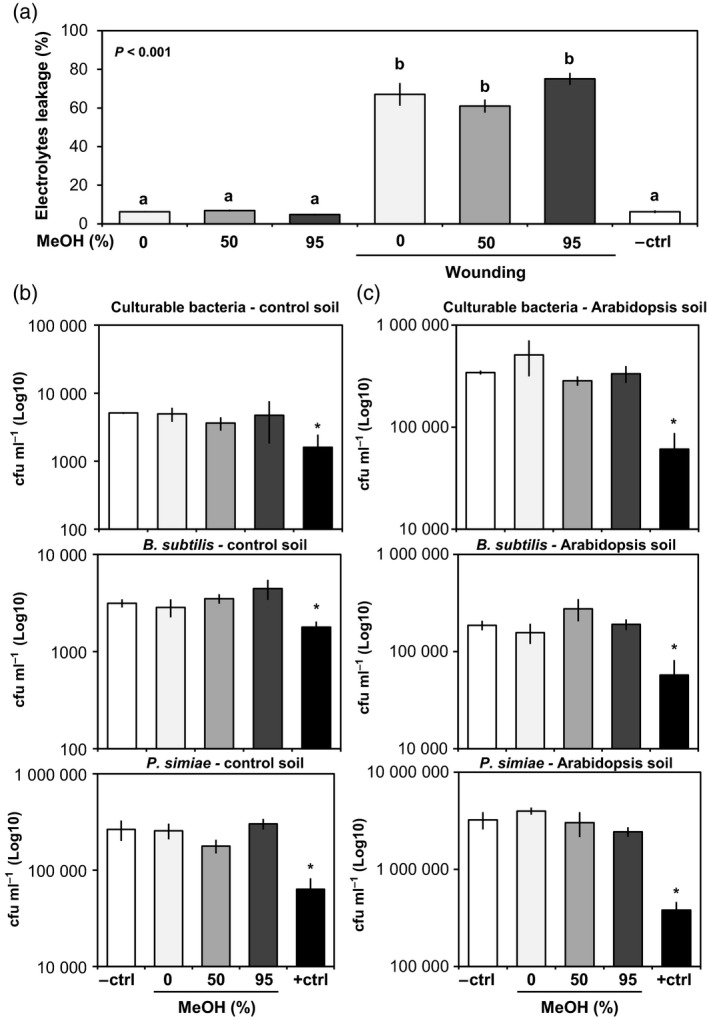
Effects of methanol (MeOH)‐containing extraction solutions on electrolyte leakage from Arabidopsis roots (a) and viability of soil microbes (b, c). (a) Quantification of electrolyte leakage from Arabidopsis roots after incubation for 1 min in acidified extraction solutions containing 0, 50 or 95% MeOH (v/v) and 0.05% formic acid (v/v). The negative control treatment (–ctrl) refers to intact roots that had not been exposed to any extraction solution. As a positive control treatment for cell damage, wounding was inflicted prior to incubation by cutting roots with a razor blade. Shown are average levels of conductivity (*n* = 4, ±SEM), relative to the maximum level of conductivity after tissue lysis (set at 100%). Statistically significant differences between treatments were determined by a Welch's *F*‐test for ranked data (*P* values indicated in the upper left corner), followed by Games–Howell *post‐hoc* tests (*P* < 0.05; different letters indicate statistically significant differences). (b, c) Effects of MeOH‐containing extraction solutions on viability of soil (b) and rhizosphere (c) microbes. Shown are average values of colony forming units (CFUs) per g of soil for culturable soil bacteria, *Bacillus subtilis* 168 and *Pseudomonas simiae* WCS417r, from extraction solution‐treated soils (*n *= 3, ±SEM). Asterisks indicate statistically significant differences between negative control (water‐flushed soil) and the corresponding treatment *(P* < 0.05, Student's *t*‐test). In all cases, only positive controls (i.e. incubation in 95% MeOH for 45 min) showed statistically significant differences.

To test whether exposure to the MeOH‐containing extraction solutions has a damaging effect on plant roots, we incubated intact roots of Arabidopsis for 1 min in acidified extraction solutions with different MeOH concentrations [0, 50 and 95% (v/v) MeOH + 0.05% (v/v) formic acid]. As a negative control, tissues were incubated for 1 min in water. To minimize root damage prior to treatment, roots were collected from agar‐grown plants. As a positive control for cell damage, tissues were wounded before incubation. After incubation, tissues were transferred to sterile water for the quantification of electrolyte leakage, which is a sensitive method to quantify cell damage in Arabidopsis (Pétriacq *et al*., 2016a). As shown in Figure 3a, none of the extraction solutions increased the level of electrolyte leakage in comparison with water‐incubated roots (Figure 3a). Hence, 1‐min exposure to the MeOH‐containing solutions does not induce ion leakage from the root cells of Arabidopsis. To investigate further the potentially damaging effects of the MeOH‐containing solutions on root cell integrity, we carried out microscopy studies. Based on the assumption that cell damage by MeOH would permeabilize root cells and cause the denaturation of cytoplasmic proteins, we used the fluorescence of a C‐terminal fusion between the cytoplasmic aspartyl‐tRNA synthetase IBI1 and YFP as a marker for root cell integrity (Luna *et al*., 2014). Roots of 2‐week‐old *35S*::*IBI1*:*YFP* plants were carefully removed from MS agar medium, incubated for 1 min in extraction solutions or water (negative control), and analysed for YFP fluorescence (Figure S4). As a positive control for cell damage, *35S::IBI1:YFP* roots were incubated for 15 min in 100% MeOH. YFP fluorescence in roots incubated in acidified 0% MeOH and 50% MeOH solutions was similar to roots incubated for 1 min in water (negative control). Some roots incubated in acidified 95% MeOH showed a weaker YFP signal, although this reduction was less severe than the near complete loss of YFP fluorescence in roots after incubation for 15 min in 100% MeOH (positive control). Thus, 1‐min exposure to the 0 and 50% MeOH solutions does not have detectable effects on root cell integrity, which is in line with our conductivity measurements (Figure 3).

To investigate whether the extraction solutions affect soil microbes, control and Arabidopsis soils were drenched for 1 min with the extraction solutions, and microbial viability was tested by dilution plating onto (non‐)selective LB agar plates. The viability of culturable soil bacteria was quantified by colony counting on non‐selective plates. To test impacts on specific rhizosphere‐colonizing bacterial strains, the Gram‐negative *Pseudomonas* (*P*.) *simiae* WCS417r (formally known as *P. fluorescens* WCS417r; Berendsen *et al*., 2012) and the Gram‐positive *Bacillus* (*B*.) *subtilis* 168 (Yi *et al*., 2016) were introduced into separate tubes 2 days prior to treatment with extraction solution, and plated onto selective agar plates after the application of extraction solution. The CFUs from solution‐treated soils were compared with water‐treated soils (1 min; negative control), as well as soils that had been treated for 45 min with 95% MeOH (positive control for microbial cell damage). Whereas the 45‐min incubation with 95% MeOH reduced bacterial counts by 10‐ to 100‐fold, none of the acidified MeOH solutions had a statistically significant effect on CFU counts from either soil type in comparison with water‐treated soil (Figure 3b,c).

In summary, our control experiments for cell damage show that 1‐min extraction with the 0 and 50% MeOH solutions does not have detectable impacts on root cell integrity and viability of soil bacteria. Direct exposure of roots to acidified 95% MeOH solution does have a minor effect on root cell integrity, however, as evidenced by the faint loss of YFP fluorescence (Figure S4). Accordingly, we cannot exclude the possibility that metabolic profiles obtained with the 95% MeOH solution are contaminated with cellular metabolites from damaged root cells.

### Untargeted metabolic profiling of control and Arabidopsis soil by UPLC‐Q‐TOF mass spectrometry

**Figure 4:**
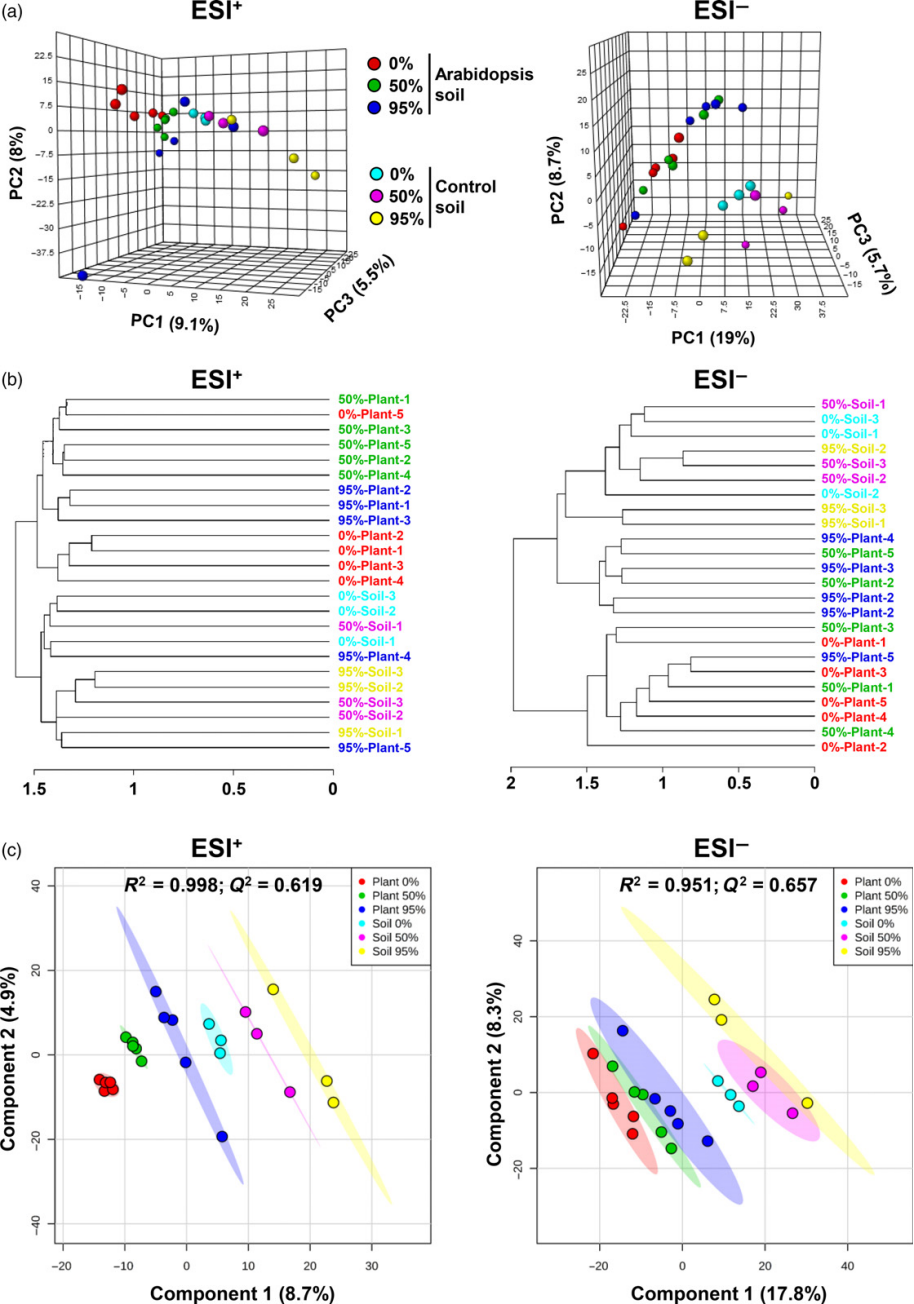
Global differences in metabolite profiles between extracts from control soil (‘soil’) and Arabidopsis soil (‘plant’). Shown are multivariate and hierarchical cluster analyses of mass spectrometry data from extracts with different extraction solutions (indicated by % MeOH). Ions (*m/z* values) were obtained by UPLC‐Q‐TOF analysis in both positive (ESI ^+^) and negative (ESI ^–^) ionization mode. Prior to analysis, data were median‐normalized, cube root‐transformed and Pareto‐scaled. (a) Unsupervised three‐dimensional principal component analysis (3D‐PCA). Shown in parentheses are the percentages of variation explained by each principal component (PC). (b) Cluster analysis (Pearson's correlation). (c) Supervised partial least squares discriminant analysis (PLS‐DA). *R* ^2^ and *Q* ^2^ values indicate the correlation and predictability values of PLS‐DA models, respectively.

Soil samples were extracted with the three acidified solutions (0.05% formic acid, v/v), containing increasing MeOH concentrations (0, 50 and 95% MeOH). Chemical profiles were obtained by untargeted UPLC‐Q‐TOF mass spectrometry (MS), using MS^E^ profiling technology (Appendix S1), which enables the simultaneous acquisition of both intact parent ions and fragmented daughter ions (Glauser *et al*., 2013; Gamir *et al*., 2014a,b; Planchamp *et al*., 2014; Pétriacq *et al*., 2016a,b). Prior to statistical analysis, chemical profiles of ion intensity were aligned and integrated using xcms (Smith *et al*., 2006; Pétriacq *et al*., 2016a,b). Similarities and differences in ion intensities from both positive (electrospray ionization source, ESI^+^, 17 518 cations) and negative (ESI^–^, 19 488 anions) ionization modes were first examined by multivariate data analysis, using metaboanalyst 3.0 (Xia *et al*., 2015). Unsupervised three‐dimensional principal component analysis (3D‐PCA) separated samples from both soil types that had been extracted with the same solution (Figure 4a), indicating global metabolic differences between control and Arabidopsis soil. These differences were reproducible between three independent experiments (Figure S5). Extractions with the 95% MeOH solution resulted in higher levels of variation than extractions with the 50% and 0% MeOH solutions (Figures 4a and S5). Cluster analysis (Pearson's correlation) revealed complete segregation between control soil samples and Arabidopsis soil samples analysed in positive ionization mode (ESI^+^), whereas samples analysed in negative ionization mode (ESI^–^) showed partial segregation between both these soil types. Although samples from the same extraction solution clustered relatively closely within the dendrogram, extracts from the 95% MeOH solution showed more variation than the other solutions (Figure 4b). Finally, we used supervised partial least squares discriminant analysis (PLS‐DA) to compare metabolite profiles between samples from control soil and Arabidopsis soil (Figure 4c). Comprehensive analysis of all samples revealed a clear separation between all different soil/solution combinations, in both the ESI^+^ and ESI^–^ data. The corresponding PLS‐DA models displayed high levels of correlation (*R* ^2^ ESI^+^_ _= 0.998; *R* ^2^ ESI^–^ = 0.951) and predictability (*Q* ^2^ ESI^+^ = 0.619; Q^2^ ESI^–^ = 0.657). Binary comparisons between control and Arabidopsis soil for each extraction solution confirmed these differences, each with high levels of correlation (*R* ^2^ > 0.94) and predictability (*Q* ^2^ > 0.59) of the PLS‐DA models (Figure S6). As was also clear from 3D‐PCA and Pearson's correlation analyses, however, samples extracted with the 95% MeOH solution were more variable than extracts obtained with the 0 and 50% MeOH solutions (Figure 4a–c). The enhanced variation between samples extracted with the 95% MeOH solution is consistent with our finding that direct exposure of roots to 95% MeOH solution causes minor cell damage (Figure S4). Together, our results show consistent differences in polar and apolar metabolite composition between control soil and Arabidopsis soil, indicating a global influence of roots on the chemical composition of the soil in our cultivation system.

### Quantitative differences in metabolites between extractions from the rhizosphere and from control soil

**Figure 5:**
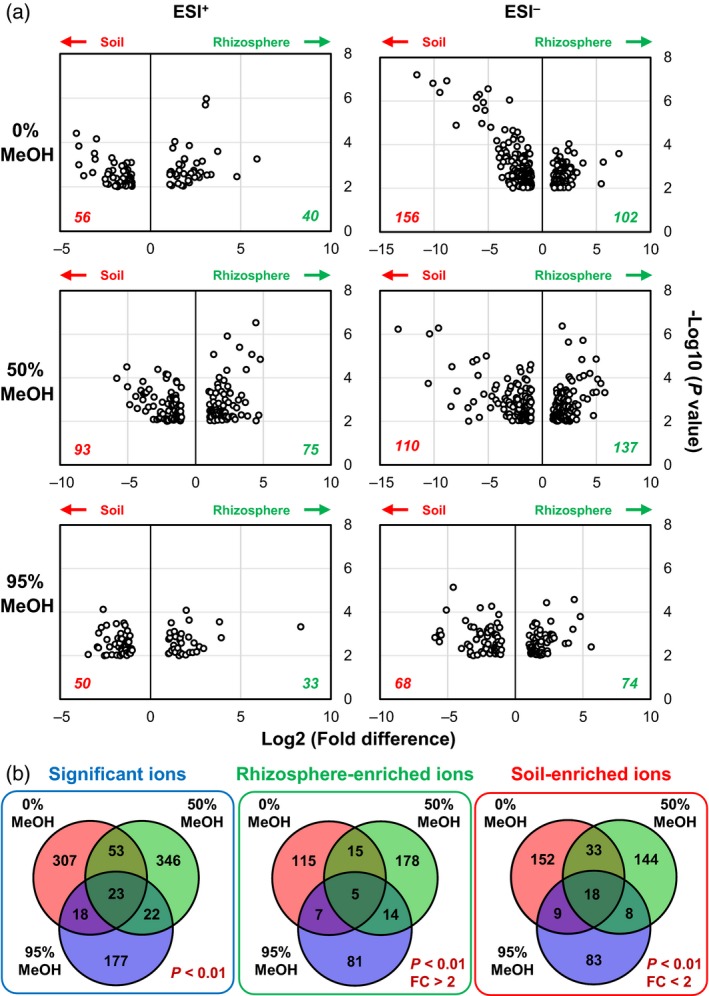
Quantitative differences in metabolite abundance between extracts from control soil and Arabidopsis soil. (a) Volcano plots expressing statistical enrichment of ions (Welch's *t*‐test) as a function of fold difference in control soil (red; ‘soil’) and Arabidopsis soil (green; ‘rhizosphere’). Data shown represent positive (ESI ^+^) and negative (ESI ^–^) ions from extractions with different solutions (indicated by % MeOH). Cut‐off values were set at *P* < 0.01 (–Log10 = 2) and fold change > 2 (Log2 = 1). (b) Venn diagrams showing overlap in ions (cations and anions combined) that significantly differ between control and Arabidopsis soil samples (left panel; *P* < 0.01, Welch's *t*–test; without fold‐change threshold), enriched in extracts from Arabidopsis soil (middle panel; >2‐fold enrichment to soil at *P* < 0.01, Welch's *t*‐test) and enriched in control soil (right panel; <2‐fold enrichment to rhizosphere at *P* < 0.01, Welch's *t*‐test).

Quantification of the total number of detected ions (*m/z* values) yielded marginally higher numbers from samples of control soil compared with that of Arabidopsis soil (Figure S7a). A substantial fraction could be detected in both soil types (66.9, 64.1 and 49.4% for the 0, 50 and 95% MeOH solutions, respectively; Figure S7a), indicating a large number of metabolites that were present in both rhizosphere and control soil. Ions that were uniquely present in one or more sample from Arabidopsis soil were most abundant in extractions with the 95% MeOH solution (6448), followed by the 50% MeOH solution (4362) and the 0% MeOH solution (3991; Figure S7a). To select for ions that were statistically over‐ or under‐represented in Arabidopsis soil, we constructed volcano plots that expressed statistical significance of each ion (*m/z* value) against fold‐change between both soil types (Figure 5a). Using a statistical threshold of *P *< 0.01 (Welch's *t*‐test) and a cut‐off value of greater than twofold change (Log2 > 1), the numbers of ions enriched in control soil were generally higher than those enriched in Arabidopsis soil (Figure 5a). Furthermore, there was relatively little overlap in differentially abundant ions between extraction solutions (*P *< 0.01, Welch's *t*‐test, Figures 5b and S7). This pattern was equally clear for ions that were specifically enriched in either soil type (*P *< 0.01, Welch's *t*‐test, greater than twofold change; Figures 5b and S7b, middle and right), illustrating the fact that the acidified solutions extracted different classes of metabolites. The 50% MeOH solution yielded the highest number of rhizosphere‐enriched ions (178), followed by the 0% MeOH solution (115) and 95% MeOH solution (81). As the 50% MeOH solution also yielded relatively low levels of variability between replicate samples (Figures 4 and S5), our results suggest that this solution is most suitable for the extraction of rhizosphere‐enriched metabolites.

### Composition of rhizosphere‐ and control soil‐enriched metabolites

**Figure 6:**
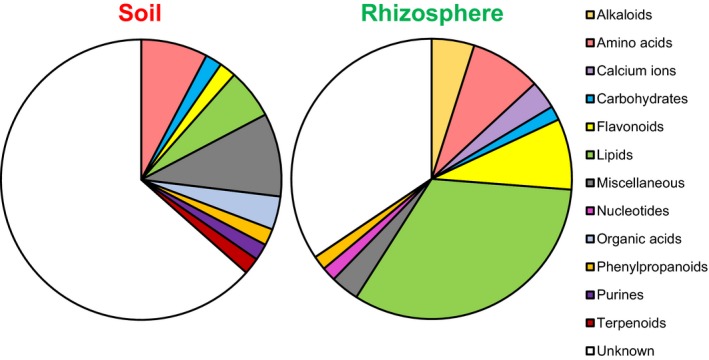
Composition of putative metabolites enriched in control soil (left) or Arabidopsis soil (right). Differentially abundant ions were selected from the top‐20 ranking ions of each volcano plot (Figure 5a) and filtered for statistical significance between all soil/extraction solution combinations (anova with Benjamini–Hochberg FDR;* P* < 0.01). The resulting 76 rhizosphere‐enriched ions and 75 control soil‐enriched ions were corrected for adducts and/or C isotopes (tolerance: *m/z* = 0.1 Da and retention time = 10 sec), and cross‐referenced against publicly available databases for putative identification. A comprehensive list of all rhizosphere‐ and soil‐enriched markers is presented in Table S1. Multiple ions putatively annotating to the same metabolite were counted additively towards the metabolite classes in the pie charts. Putative metabolites that are unlikely to accumulate as natural products in (rhizosphere) soil (e.g. synthetic drugs or mammalian hormones) were not included in the final selection presented. Miscellaneous: putative metabolites that do not belong to any of the other metabolite classes listed. Unknown: ion markers that could not be assigned to any known compound.

To study which metabolite classes drive the global differences between the rhizosphere and control soil (Figures 4 and 5), we pooled the top‐20 ranking ions from each volcano plot, ranked by fold change and statistically significant difference between control and Arabidopsis soil, resulting in a total of 120 metabolic markers for each soil type. To enhance statistical stringency, ions were subsequently filtered by statistical significance between all soil/solution combinations (anova;* P* < 0.01), using a Benjamini–Hochberg correction for false‐discovery rate (FDR). The final selection yielded a total of 76 rhizosphere‐enriched ions and 75 control soil‐enriched ions. marvis (Kaever *et al*., 2012) was used to correct for adducts and/or C isotopes (tolerance: *m/z *= 0.1 Da and retention time = 10 sec), after which the predicted masses were used for putative identification (Table S1), using METLIN, PubChem, MassBank, Lipid Bank, ChemSpider, Kegg, AraCyc and MetaCyc databases (Kaever *et al*., 2009, 2012; Gamir *et al*., 2014a,b; Pastor *et al*., 2014; Pétriacq *et al*., 2016a,b). To obtain a global profile of soil‐ and rhizosphere‐enriched chemistry, putative compounds were assigned to different metabolite classes (Figure 6). Putative chemicals that are unlikely to accumulate as natural products in (rhizosphere) soil, such as synthetic drugs or mammalian hormones, were excluded from these profiles (Table S1). In comparison with control soil, Arabidopsis soil was enriched with ions that putatively annotate to flavonoids (8 versus 2%), lipids (33 versus 6%) and alkaloids (5% in Arabidopsis soil only; Figures 6 and S8; Table S1), which supports the notion that rhizosphere soil is enriched with plant‐derived metabolites. The global composition of control soil showed a higher fraction of metabolites that could not be annotated (Figures 6 and S8; Table S1), probably because of an under‐representation of soil metabolites in publically available databases.

### Applicability of the method to maize in agricultural soil

**Figure 7:**
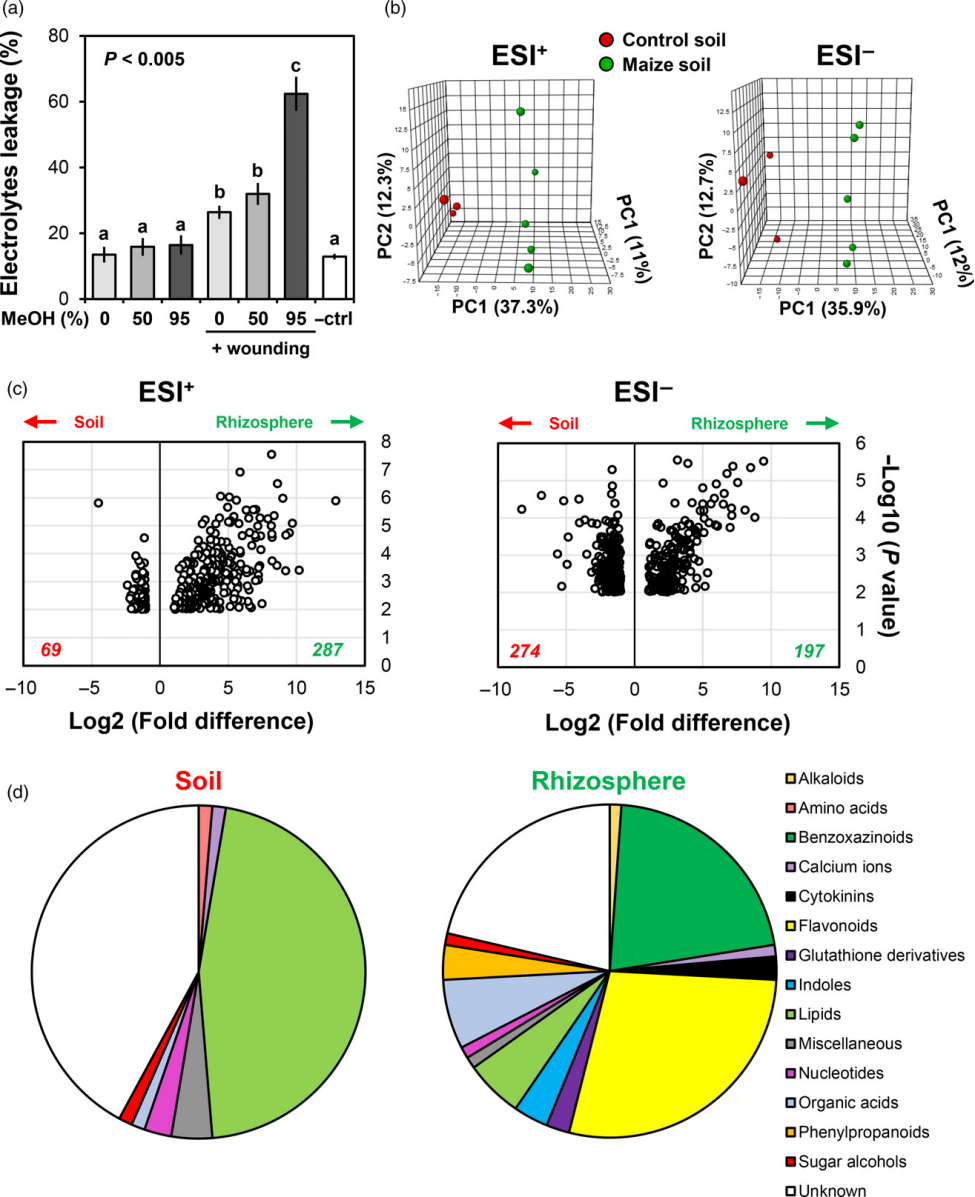
Applicability of the profiling method for maize in agricultural soil. The experimental system for extracting soil chemistry was based on 50‐mL collection tubes filled with a mixture of agricultural soil from arable farmland and perlite (75 : 25, v/v). Samples were extracted with the 50% MeOH (v/v) solution 17 days after planting. (a) Quantification of maize root damage after direct exposure to the extraction solutions. Five‐day‐old maize roots were incubated for 1 min in acidified extraction solutions containing 0, 50 or 95% MeOH (v/v) and tested for electrolyte leakage by conductivity. For details, see the legend to Figure 3a. Shown are average levels of conductivity (*n* = 4, ±SEM), relative to the maximum level of conductivity after tissue lysis (set at 100%). Statistically significant differences between treatments were determined by a Welch's *F*‐test for ranked data (*P* values indicated in the upper left corner of each panel), followed by Games–Howell post‐hoc tests (*P* < 0.05; different letters indicate statistically significant differences). (b) Unsupervised 3D‐PCA, showing global differences in metabolic profiles between control soil (red) and maize soil (green). Shown are data from extracts with the 50% MeOH (v/v) extraction solution. For further details, see the legend to Figure 4. (c) Volcano plots expressing statistical enrichment of ions (Welch's *t*‐test) as a function of fold difference in control soil (red; ‘soil’) and maize soil (green; ‘rhizosphere’). Cut‐off values were set at *P* < 0.01 (–Log10 = 2) and fold change > 2 (Log2 = 1). (d) Relative composition of putative metabolite classes enriched in control soil (left) or maize soil (right). Differentially abundant metabolites were selected from the top‐50 ranking ions of each volcano plot (ESI^+^ and ESI^–^; c), corrected for adducts and/or C isotopes (tolerance: *m/z* = 0.1 Da and retention time = 10 s), and cross‐referenced against publicly available databases for putative identification. A comprehensive list of all rhizosphere‐ and soil‐enriched markers is presented in Table S2. Putative metabolites that unlikely accumulate as natural products in (rhizosphere) soil (e.g. synthetic drugs or mammalian hormones) were not included in the final selection presented. For further details, see the legend to Figure 6.

Having established that our method is suitable for detecting rhizosphere‐enriched metabolites from Arabidopsis, we investigated whether the method could be applied to profile rhizosphere metabolites from a crop species (maize) in agricultural soil. To this end, the cultivation system was up‐scaled to 50‐ml tubes that were filled with a mixture of agricultural soil from arable farmland (Spen Farm, Leeds, UK) and perlite (75 : 25, v/v). The perlite was added to improve the drainage of the soil, which improved plant growth and ensured that sufficient solution was collected from the base of the tubes within 1 min of the application of extraction solution. Maize plants were grown for 17 days, and rhizosphere chemistry was extracted using the 50% MeOH solution (plus formic acid 0.05%, v/v). Further validation experiments showed that 1‐min exposure of maize roots to this solution did not lead to increased electrolyte leakage (Figure 7a). Comparative analysis of metabolites by UPLC‐Q‐TOF identified a total of 6071 cations (ESI^+^) and 9006 anions (ESI^–^). 3D‐PCA showed complete separation between samples from control (red) and maize (green) soil (Figure 7b). Quantitative differences were determined by volcano plots (Welch's *t*‐test, *P* < 0.01: fold change > 2), revealing 287 cations (ESI^+^) and 197 anions (ESI^–^) that were statistically enriched in maize soil (Figure 7c). Cross‐referencing the 100 most significant ions (top 50 anions plus top 50 cations) against public databases indicated higher levels of chemical diversity in maize soil samples compared with control soil samples. Most metabolic markers could be putatively identified (Table S2) and annotated to different metabolite classes (Figure 7d). As described for the profiling of the Arabidopsis rhizosphere (Figure 6), these final profiles did not include putative compounds that are unlikely to accumulate in (rhizosphere) soil, such as synthetic drugs (Table S2). Strikingly, a relatively large fraction of maize rhizosphere‐enriched ions could be annotated to flavonoids (28%) and benzoxazinoids (21%), which mediate below‐ground interactions (Neal *et al*., 2012; Robert *et al*., 2012; Neal and Ton, 2013) For instance, HBOA, DIBOA and HMBOA displayed strong rhizosphere enrichment in maize soil samples (Figure S8), and are known to be produced by maize roots (Marti *et al*., 2013). Thus, our profiling method is sufficiently robust and sensitive to profile plant‐derived rhizosphere chemicals from a crop species in agricultural soil.

### Profiling chemistry in distal rhizosphere fractions

The rhizosphere was defined by Lorenz Hiltner in 1904 as ‘the soil compartment influenced by the root’ (Smalla *et al*., 2006); however, many rhizosphere studies focus exclusively on soil that is closely associated with plant roots (after the removal of loosely associated soil), which may not encompass the total rhizosphere as more distal and loosely associated soil could still be influenced by root‐derived chemistry. To investigate whether our profiling method detects chemical influences beyond soil that is closely associated with roots, we used an alternative growth system that separated roots from distal soil (Figure S9). Maize plants were grown in small, fine mesh bags within larger 150‐ml tubes containing soil (see Appendix S1), which prevented outward root growth, yet allowed for the passage of root‐derived chemicals and microbes into the distal soil. Similar plant‐free tubes were constructed as no‐plant controls. After 24 days of growth the mesh bags were carefully removed, and then metabolites were extracted from the remaining distal soil that had surrounded the mesh bags using the 50% MeOH extraction solution. As a control for whole‐soil fractions, metabolites from empty and maize‐containing tubes were extracted before removing the mesh bag from the tube, as described earlier. Thus, the experimental design allowed for comparison between four soil fractions: (i) distal soil surrounding mesh bags without roots; (ii) distal soil surrounding mesh bags with maize roots; (iii) whole soil from tubes containing mesh bags without roots; and (iv) whole soil from tubes containing mesh bags with maize roots. Extracts were analysed by UPLC‐Q‐TOF in ESI^–^ (26 011 anions) and subjected to unsupervised PCA (Figure S9b). Comparison of whole‐soil fractions confirmed a clear separation between plant‐free and maize soil samples, illustrating the chemical rhizosphere effect of maize. Although less pronounced than the whole‐soil fractions, PCA of the distal soil fractions still revealed separate clustering between plant‐free and maize soil (Figure S9b), indicating that the chemical influence of the rhizosphere extended beyond the soil closely associated with roots. To verify this distant rhizosphere effect, we quantified levels of DIMBOA, which acts as a relatively stable rhizosphere semiochemical, influencing the behaviour of both rhizobacteria and arthropods (Neal *et al*., 2012; Robert *et al*., 2012). In comparison to both plant‐free soil fractions, statistically higher quantities of DIMBOA were detected in both whole maize soil and distal maize soil (Figure S9c). Hence, DIMBOA acts as a mobile long‐range rhizosphere signal that extends beyond soil that is closely associated with roots. Considering that maize roots contain high quantities of DIMBOA (Robert *et al*., 2012), and that the distal soil was separated from the roots prior to chemical extraction with the 50% MeOH solution, this result also confirms that the 50% MeOH extraction solution does not have a damaging effect on maize roots, as exemplified by similar DIMBOA levels in whole maize soil and distal maize soil (Figure S9c).

## Discussion

Rhizosphere chemistry is a complex mixture of root exudation chemicals, their microbial breakdown products and the microbial breakdown products of soil‐specific chemicals. Although it is known that microbial diversity in the rhizosphere can influence plant growth and health (Berendsen *et al*., 2012), the chemical signals mediating these interactions remain poorly understood. The majority of root exudation studies are based on hydroponic and/or sterile growth systems (Khorassani *et al*., 2011; Kuijken *et al*., 2014; Bowsher *et al*., 2016). Although sterile growth systems are appropriate for the exact quantification of root‐exuded plant chemicals (Kuijken *et al*., 2014), these systems do not consider the importance of rhizosphere signals that are of microbial origin, such as microbial breakdown products of root exudates, or metabolites that are specifically produced by rhizosphere‐inhabiting microbes. Consequently, linking rhizosphere chemistry with microbial communities and/or activities remains problematic when the biochemical diversity of the non‐sterile rhizosphere is not considered (Oburger and Schmidt, 2016). Furthermore, although root exudation studies are increasingly relying on sensitive analytical methods (Khorassani *et al*., 2011; Ziegler *et al*., 2015; van Dam and Bouwmeester, 2016), the majority of these studies employ targeted analyses of specific compounds (e.g. organic and amino acids, coumarins) that do not address the biochemical diversity of rhizosphere soil. Recent advances in liquid chromatography, mass spectrometry, and uni‐ and multivariate data analysis have made it possible to conduct untargeted metabolic profiling of complex metabolite mixtures, such as root exudates and soil extracts (Khorassani *et al*., 2011; Strehmel *et al*., 2014; Swenson *et al*., 2015; Ziegler *et al*., 2015; van Dam and Bouwmeester, 2016). In this study, we employed untargeted UPLC‐Q‐TOF analysis of soil extracts, followed by uni‐ and multivariate data reduction to separate rhizosphere‐specific chemistry from common soil chemistry. We show that this method is suitable to profile *in situ* rhizosphere chemistry from different plant species and soil types.

The microbial rhizosphere effect is driven by root exudation chemistry (Jones *et al*., 2009). Accordingly, we verified whether our cultivation system supported the generation of a difference in microbial communities between control soil samples (without plant roots) and root samples plus adhering rhizosphere soil, using *16S* rRNA gene sequencing. This analysis identified a total number of 3863 OTUs, which by rarefaction analysis appeared to be sufficient to cover the majority of dominant OTUs (Figure S1). Many of the taxa detected in our samples (e.g. Oxalobacteraceae, Pseudomonadaceae, Xanthomonadaceae and the Rhizobiaceae) are commonly associated with soil and/or plant roots (Lundberg *et al*., 2012). Comparative analysis identified a range of OTUs with differential relative abundance between control soil and ‘root plus rhizosphere’ samples (Figure 2), which provided evidence for a rhizosphere effect in our experimental growth system. Many of the corresponding taxa have been linked to rhizosphere effects, such as an enhanced relative abundance of Oxalobacteraceae (Figures 2b and S2; Lundberg *et al*., 2012; Bulgarelli *et al*., 2015), as well as the Rhizobiales, which are commonly associated with plant roots (Hao *et al*., 2016).

Our cultivation system was designed for the *in situ* extraction of chemicals from biologically complex non‐sterile rhizosphere soils. The soil matrix for the Arabidopsis experiments consisted of a 9 : 1 (v/v) mixture of sand and compost, which is comparable with the sandy soil types of naturally occurring Arabidopsis accessions (Lev‐Yadun and Berleth, 2009). This matrix also allowed relatively short collection times of the extracts (1 min), which was sufficient to recover 90% of the volume applied and prevents root damage through extended exposure to MeOH in the extraction solution. The soil matrix for the maize experiments contained agricultural soil from an arable farm field, which was supplemented with 25% (v/v) autoclaved perlite to prevent compaction, and allowed sufficient elution of metabolites over the 1‐min extraction period. Using this system, we detected quantitative and qualitative differences in chemistry between extracts from control and maize soil (Figure 7), demonstrating that the method was applicable for the profiling of rhizosphere chemistry from a crop species in agricultural soil.

A major challenge for the *in situ* profiling of rhizosphere chemistry is to prevent damage of root cells and microbes during the extraction procedure that could otherwise contaminate the extract with metabolites that are not exuded from intact roots. Whereas water‐based extraction solutions are unlikely to cause cellular damage, they are less suitable for the extraction of apolar metabolites. Conversely, solutions containing organic solvents extract apolar metabolites, but can damage cell membranes. With our limited understanding of root exudation chemistry in natural soil types, it remains difficult to distinguish between naturally exuded metabolites and metabolites leaking from damaged root tissues or lysed microbial cells. Therefore, we carried out a range of experiments to investigate whether the MeOH‐containing extraction solutions caused cell damage: (i) quantification of root electrolyte leakage (Figure 3a); (ii) epifluorescence microscopy, to assess root cell integrity (Figure S4); (iii) dilution plating, to assess the viability of soil‐ and rhizosphere‐colonising bacteria after incubation of the soil in extraction solutions (Figure 3b, c); and (iv) detection of plant‐derived chemicals in root‐free soil fractions (Figure S9). Firstly, exposure of both Arabidopsis and maize roots to the MeOH‐containing solutions did not increase electrolyte leakage for the duration of the extraction procedure (1 min; Figures 3 and 7). Secondly, microscopic analysis of root cells from YFP‐expressing Arabidopsis roots did not reveal any loss of cell integrity after 1 min of exposure to 0 and 50% MeOH‐containing solutions (Figure S4). This assay did reveal a weak effect of the 95% MeOH solution, however, indicating that extraction of rhizosphere chemistry with this solution could affect root cell integrity. Thirdly, the extraction of control and Arabidopsis soil with the MeOH‐containing extraction solutions did not reduce the viability of culturable soil microbes, nor did it affect the viability of the Gram‐negative rhizobacterial strain *P. simiae* WCS417r and the Gram‐positive rhizobacterial strain *B. subtilis* 168 (Figure 3b,c). Finally, using the 50% MeOH extraction solution and a compartmentalized growth system that separated maize roots from peripheral rhizosphere soil, we showed that the extraction of peripheral soil after the removal of maize roots yielded similar DIMBOA quantities as the extraction of soil containing maize roots (Figure S9c). As maize roots accumulate high quantities of DIMBOA (Robert *et al*., 2012), this result further confirms that the 50% MeOH extraction solution does not damage maize roots in the soil. Accordingly, we conclude that 1 min of exposure to 0 or 50% MeOH extraction solution does not cause detectable levels of cell damage to roots and soil microbes that could contaminate the chemical profiles from the soils with intracellular metabolites.

Multivariate data analysis and clustering revealed that the variability between replicate extractions was lower for the 0 and 50% MeOH extraction solutions, compared with the 95% MeOH solution (Figure 4). This is consistent with our finding that direct exposure to this solution sometimes reduced YFP fluorescence in transgenic Arabidopsis roots (Figure S4). Data projection in volcano plots showed that extraction with the 50% MeOH solution yielded the highest number of rhizosphere‐enriched ions, in comparison with other extraction solutions (Figure 5a). Hence, the 50% MeOH extraction solution performs best in terms of variability between extractions and total numbers of differentially detected ions. Quantitative analysis of MS profiles revealed slightly lower numbers of rhizosphere‐enriched ions than control soil‐enriched ions, which was apparent for both Arabidopsis (Figures 5 and S7) and maize (Figure 7). It is possible that this difference arises from the rhizosphere effect, which reduces bacterial richness (Figures S1 and S2), resulting in lower biochemical diversity in the rhizosphere (Prithiviraj *et al*., 2007).

The sets of ions enriched in control and plant‐containing soil differed substantially in composition (Figures 6 and 7). Interestingly, the number of ions annotated to putative metabolites from publicly available databases was higher for rhizosphere selection (Tables S1 and S2). We attribute this difference to the fact that plant‐containing soil is enriched with plant‐derived metabolites, which are better represented in publicly available databases than soil‐specific metabolites (Strehmel *et al*., 2014; Swenson *et al*., 2015). Indeed, the selection of putative rhizosphere metabolites from Arabidopsis contained a relatively high fraction of flavonoids, lipids and other amino acid‐derived secondary metabolites, such as alkaloids and phenylpropanoids (Figure 6; Table S1), whereas the set of putative rhizosphere metabolites from maize included relatively large fractions of flavonoids and benzoxazinoids (Figure 7; Table S2). It should be noted, however, that the analytical method used in this study is limited by the putative identification of single ions. Unless the identity of a single metabolite is confirmed by subsequent targeted analyses, such as specific chromatographic retention time, fragmentation or NMR patterns, its annotation remains putative (i.e. inconclusive). The novelty of our method does not come from the applied mass spectrometry detection method, however, but from the combined use of the experimental design, extraction methods, mass spectrometry profiling and statistical techniques to deconstruct rhizosphere chemistry. Once a wider profile of rhizosphere chemistry has been established, targeted techniques can be used to confirm metabolite identities. Furthermore, where multiple putative metabolites annotate to the same metabolite class, a more reliable conclusion can be drawn about the involvement of this metabolite class. In our case, multiple rhizosphere ions could be annotated to the same plant metabolic pathways, suggesting that the overall rhizosphere profile is influenced by these plant metabolite classes. In support of this, previous studies have reported the presence of the same secondary compounds in plant root exudates (Hassan and Mathesius, 2012; Oburger *et al*., 2013; Oburger and Schmidt, 2016; Szoboszlay *et al*., 2016). Moreover, benzoxazinoids, such as DIMBOA, have previously been implicated to act as below‐ground semiochemicals during maize–biotic interactions (Neal *et al*., 2012; Robert *et al*., 2012; Marti *et al*., 2013). Hence, our method provides a new tool to explore rhizosphere semiochemicals for different plant species and soils.

Relatively few rhizosphere‐enriched ions could be annotated to primary plant metabolites, such as proteinogenic amino acids or organic acids (Figures 6 and 7). Although these compounds are exuded in high quantities by roots (Rudrappa *et al*., 2008; Ziegler *et al*., 2015; van Dam and Bouwmeester, 2016), the microbial activity in the rhizosphere will quickly metabolize them, and the C18‐UPLC separation is not optimal for the separation of (often very polar) primary metabolites. Above all, we stress that our method is not suitable for quantitative analysis of primary and secondary root exudates, for which sterile root cultivation systems are more appropriate (Kuijken *et al*., 2014; Strehmel *et al*., 2014). Our method should only be used for the profiling, identification and/or quantification of rhizosphere chemicals. These compounds can be microbial breakdown products of secondary metabolites in root exudates, but could equally well newly synthesized by rhizosphere‐specific bacterial and fungal microbes. Using the experimental pipeline detailed in this paper, stable isotope labelling of plant root exudates via leaf exposure to ^13^CO_2_ can potentially differentiate between these classes of rhizosphere metabolites, where plant‐derived breakdown products are likely to retain higher levels of ^13^C than newly synthesized microbial products. Furthermore, as is illustrated by our study, the method allows for the simultaneous assessment of rhizosphere chemistry and microbial composition, which can be used for genetic strategies that aim to establish a causal relationship between plant genotype, rhizosphere chemistry and microbial composition (Oburger and Schmidt, 2016). Such an approach would also advance studies on the effects of above‐ground stimuli [such as light, atmospheric CO_2_ and above‐ground (a)biotic stresses] on below‐ground plant–microbe interactions.

In summary, our study presents a straightforward method to obtain profiles of rhizosphere chemistry in non‐sterile rhizosphere soil. The method is applicable to both model systems and soil‐grown crops in agricultural soil. Considering that the microbial interactions in the rhizosphere can have both beneficial and detrimental effects on plant performance (Berendsen *et al*., 2012; Cameron *et al*., 2013), our method provides a powerful tool to advance rhizosphere biology and to decipher the chemistry driving plant–microbe interaction in complex non‐sterile soils.

## Experimental Procedures

### Chemicals and reagents

All chemicals and solvents used for metabolomics were of mass spectrometry grade (Sigma‐Aldrich, https://www.sigmaaldrich.com). Other solvents were of analytical grade.

### Experimental set‐up of growth system

Collection tubes for the Arabidopsis experiments were constructed by melting 7‐mm holes in the base of 30‐ml plastic tubes (Sterilin 128A; ThermoFisher Scientific, https://www.thermofisher.com), using a soldering iron (Figure 1). The drainage hole was covered with 4‐cm^2^ pieces of Millipore miracloth (pore size, 22–25 μm, VWR, https://uk.vwr.com) to avoid any loss of soil and to prevent outgrowth by roots. Tubes were filled with ~45 g of soil matrix, consisting of a homogenous 9 : 1 (v/v) mixture of sand (silica CH52) and dry compost (Levington M3), which is comparable with the sandy soil types of naturally occurring *A. thaliana* (Arabidopsis) accessions (Lev‐Yadun and Berleth, 2009). To prevent cross contamination of rhizosphere microbes and chemicals between samples, each collection tube was placed onto an individual Petri dish (Nunclon™ Delta, 8.8 cm^2^; ThermoFisher Scientific; Figure 1). Collection tubes were wrapped in aluminium foil to limit algal growth in the soil matrix. Seeds of Arabidopsis accession Columbia (Col‐0) were stratified for 2 days in the dark in autoclaved water at 4°C. Three or four seeds were pipetted onto individual tubes and placed into a growth cabinet (Fitotron; SANYO, http://sanyo-av.com) with the following growth conditions: 8.5 h light/15.5 h dark at 21/19°C, with an average of 120 μmol m^−2^ s^−1^ photons at the top of the collection tubes and a relative humidity of 70%. Four days later seedlings were removed to leave one seedling per pot, which was grown for 5 weeks until sampling. All pots were watered twice per week by applying 5 ml of autoclaved distilled water to the Petri dishes, using a 5‐ml pipette (Starlab, https://www.starlabgroup.com). The final watering date was set at 3 days before sampling, which resulted in consistent soil water contents at the time of sampling. The relative water content (RWC) was determined by the ratio of soil weight (W) minus soil dry weight (DW), divided by water‐saturated soil weight (SW) minus soil dry weight: RWC = (W − DW)/(SW − DW).

The watering regime applied provided reproducible RWC values at the time of sampling (88 ± 4.5%). Although the RWC during the cultivation of plants was frequently lower, the relatively high RWC value at the time of sampling allowed for constant and relatively high recovery volumes (4–4.5 ml) from the soil matrix.

Collection tubes for the maize experiments were constructed by melting 7‐mm holes in the base of 50‐ml plastic tubes. Tubes were fitted with Miracloth at the bottom and filled with a water‐saturated mixture of agricultural soil/autoclaved perlite (75 : 25; v/v), in order to allow for a sufficient collection volume 1 min after the application of extraction solutions (see below). Soil was collected from an arable field (Spen Farm, Leeds, UK), air‐dried, sieved to a maximum particle size of 4.75 mm and homogenized using a mixer. Maize seeds (*Z. mays* variety W22) were surface sterilized for 3 h by placing them in Petri dishes in an airtight container with 100 ml of bleach, to which 5 ml of concentrated HCl had been added. Seeds were imbibed overnight in autoclaved, sterile water before placing on Petri dishes containing sterile, damp filter paper in the dark at 23°C for 2 days. Germinated seeds were planted in filled collection tubes, 1.5 cm from the soil surface. Collection tubes were wrapped in foil, covered with black plastic beads and placed in a growth chamber with the following conditions: 12 h light/12 h dark at 25/20°C. The additional maize experiment to profile distal rhizosphere chemistry is described in Appendix S1.

### Profiling of root associated microbial communities

Details about DNA extraction, *16S* rRNA gene sequencing and analysis of root‐associated prokaryotic OTUs are presented in Appendix S1.

### Metabolite extraction from control and Arabidopsis/maize soil

Plant soil samples were collected from tubes containing one 5‐week‐old Arabidopsis plant or one 17‐day‐old maize plant. Plant soil chemistry was analysed from five replicated samples, whereas control soil chemistry was analysed from three replicated samples. All samples were collected at the same time. For the Arabidopsis system, cold extraction solution (5 ml) containing 0, 50 or 95% methanol (v/v) with 0.05% formic acid (v/v) was applied to the top of the tubes. After 1 min, 4.0–4.5 ml was collected from the drainage hole in 5‐ml centrifuge tubes (Starlab). For the maize system, 15 ml of the 50% methanol solution (0.05% formic acid, v/v) was applied and flushed through the soil by applying pressure to the top of the pot, using a modified lid containing a syringe. After 1 min, 10 ml was collected in centrifuge tubes. For both cultivation systems, extracts were centrifuged to pellet soil residues (5 min, 3500 ***g***), after which 4 ml of supernatant was transferred into a new centrifuge tube and flash‐frozen in liquid nitrogen, freeze‐dried for 48 h until complete dryness (Modulyo benchtop freeze dryer; Edwards, https://www.edwardsvacuum.com), and stored at −80°C. Dried aliquots were re‐suspended in 100 μl of methanol : water : formic acid (50 : 49.9 : 0.1, v/v), sonicated at 4°C for 20 min, and vortexed and centrifuged (15 min, 14 000 ***g***, 4°C) to remove potential particles that could block the UPLC column. Final supernatants (80 μl) were transferred into glass vials containing a glass insert prior to UPLC‐Q‐TOF analysis.

### Assessment of cell damage by extraction solutions

The effects of acidified extraction solutions on the integrity of root cells were determined by conductivity measurement from electrolyte leakage and epifluorescence microscopy of transgenic YFP‐expressing roots, as detailed in Appendix S1. The effects of extraction solutions on culturable soil bacteria and introduced soil‐ and rhizosphere‐colonising bacteria were determined by dilution plating, as described in Appendix S1.

### UPLC‐Q‐TOF analysis of soil chemistry

Details of the UPLC‐Q‐TOF analysis, including the targeted detection of DIMBOA, and uni‐ and multivariate data analyses to deconstruct rhizosphere chemistry, are presented in Appendix S1.

## Accession Numbers

The sequences used in this study can be found in the European Nucleotide Archive (http://www.ebi.ac.uk/ena) under accession number PRJEB17782.

## Supporting information


**Figure S1.** Rarefaction curves of *16S* rRNA operational taxonomic units (OTUs).

**Figure S2.** Relative abundance of bacterial taxa.

**Figure S3.** Solvent polarity and extraction of rhizosphere chemistry.

**Figure S4.** Epifluorescence microscopy analysis of Arabidopsis root cell damage.

**Figure S5.** Reproducibility of metabolite profiles between experiments.

**Figure S6.** Binary PLS‐DA analysis of metabolite profiles.

**Figure S7.** Details of quantitative differences in metabolites.

**Figure S8.** Relative quantities of benzoxazinoids.

**Figure S9.** Profiling distal rhizosphere chemistry.

**Table S1.** Putative identification of Arabidopsis metabolic markers.

**Table S2.** Putative identification of maize metabolic markers.

**Appendix S1.** Supplementary experimental procedures.
