# Supplementary material for: Metabolite profiling of non‐sterile rhizosphere soil
Source: Plant J. 2017 Aug 31;92(1):147–62. doi: 10.1111/tpj.13639 (PMC5639361; doi:10.1111/tpj.13639)
Supplement: Supplementary file 1 — Figure S1. Rarefaction curves of 16S rRNA operational taxonomic units (OTUs). [file TPJ-92-147-s001.pdf]

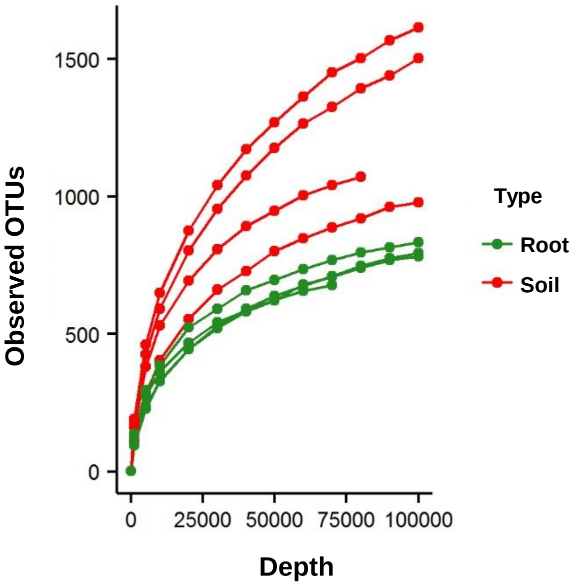


**Supplemental Figure S1.** Rarefaction curves of detected OTUs.

Shown are curves after removal of singletons for replicate root + rhizosphere samples (green) and control soil samples (red).
