# Supplementary material for: Metabolite profiling of non‐sterile rhizosphere soil
Source: Plant J. 2017 Aug 31;92(1):147–62. doi: 10.1111/tpj.13639 (PMC5639361; doi:10.1111/tpj.13639)
Supplement: Supplementary file 2 — Figure S2. Relative abundance of bacterial taxa. [file TPJ-92-147-s002.pdf]

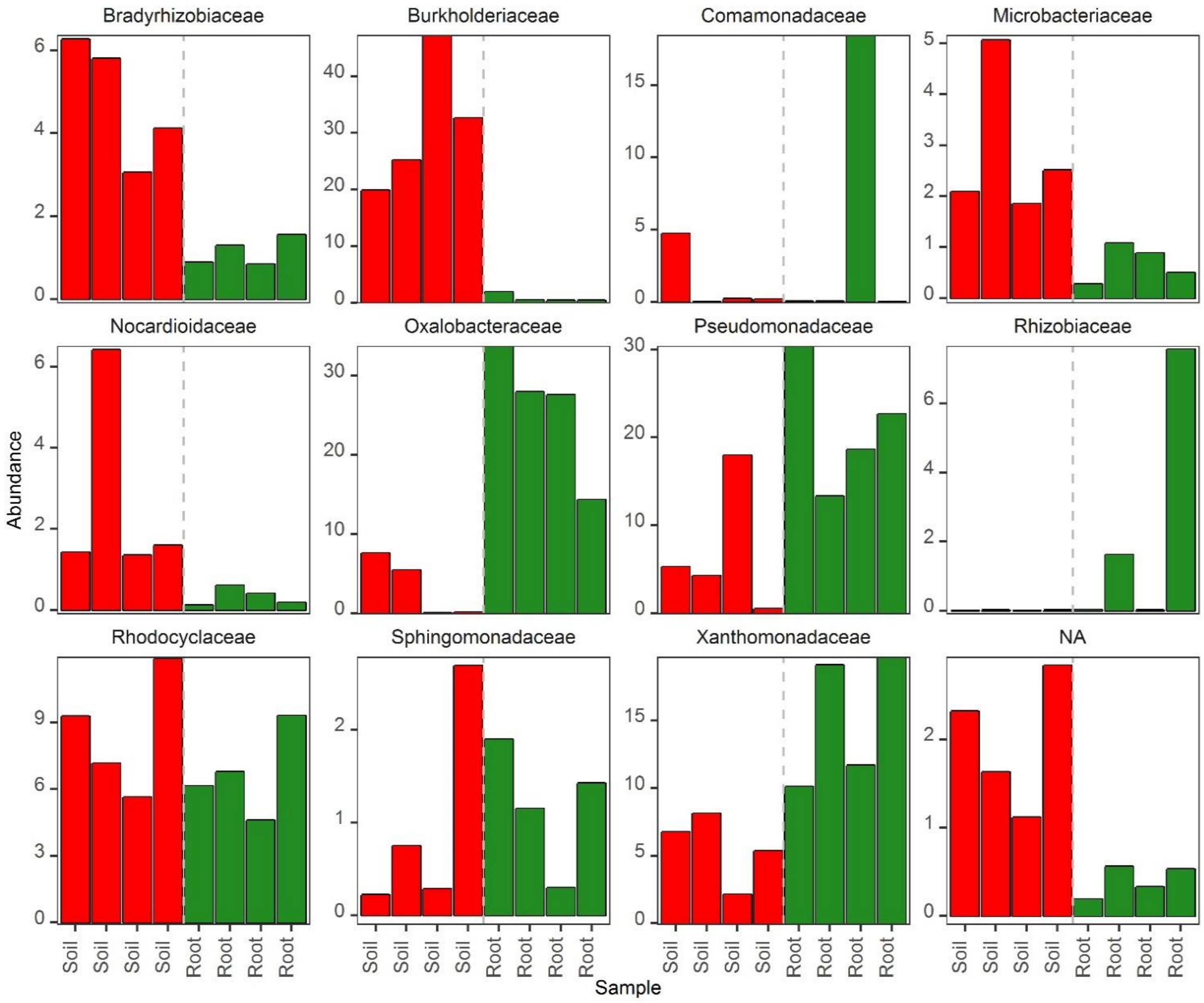

Supplemental Figure S2. Relative abundance (%) of selected families in control soil samples ('Soil'; red) and root + rhizosphere samples ('Root'; green) from the Arabidopsis growth system.

Shown are families containing OTUs with relative abundances > 2% in one or more samples. Each bar represents an individual biological replicate. NA, taxonomy not available.
