# Supplementary material for: Metabolite profiling of non‐sterile rhizosphere soil
Source: Plant J. 2017 Aug 31;92(1):147–62. doi: 10.1111/tpj.13639 (PMC5639361; doi:10.1111/tpj.13639)
Supplement: Supplementary file 3 — Figure S3. Solvent polarity and extraction of rhizosphere chemistry. [file TPJ-92-147-s003.pdf]

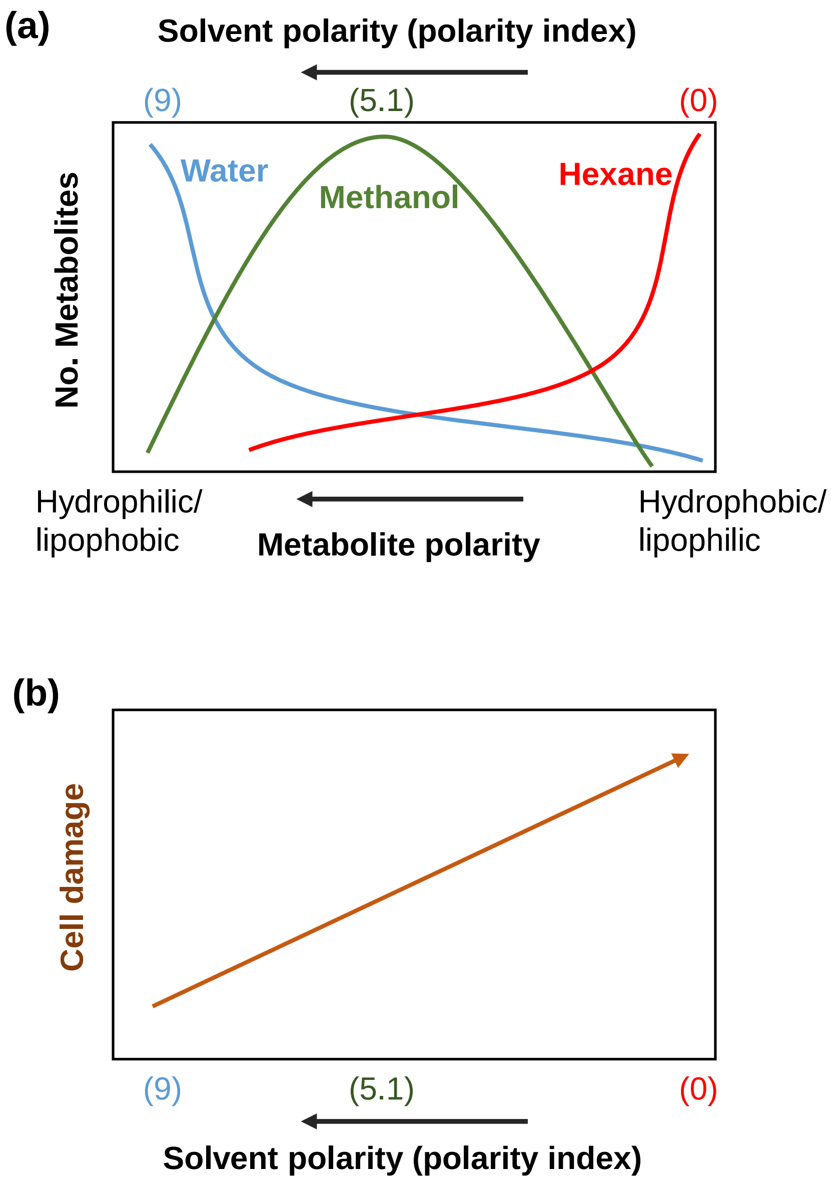

Supplemental Figure S3. Model of expected impacts of solvent polarity on the extraction of soil metabolites.

(**a**) Examples of solvent polarities and their impact on the type of metabolites extracted. Polarity index of water, methanol (MeOH) and hexane are shown within parentheses.
(**b**) Hypothesized impact of solvent polarity on cell damage of plant roots and soil microbes.
