# Supplementary material for: Metabolite profiling of non‐sterile rhizosphere soil
Source: Plant J. 2017 Aug 31;92(1):147–62. doi: 10.1111/tpj.13639 (PMC5639361; doi:10.1111/tpj.13639)
Supplement: Supplementary file 4 — Figure S4. Epifluorescence microscopy analysis of Arabidopsis root cell damage. [file TPJ-92-147-s004.pdf]

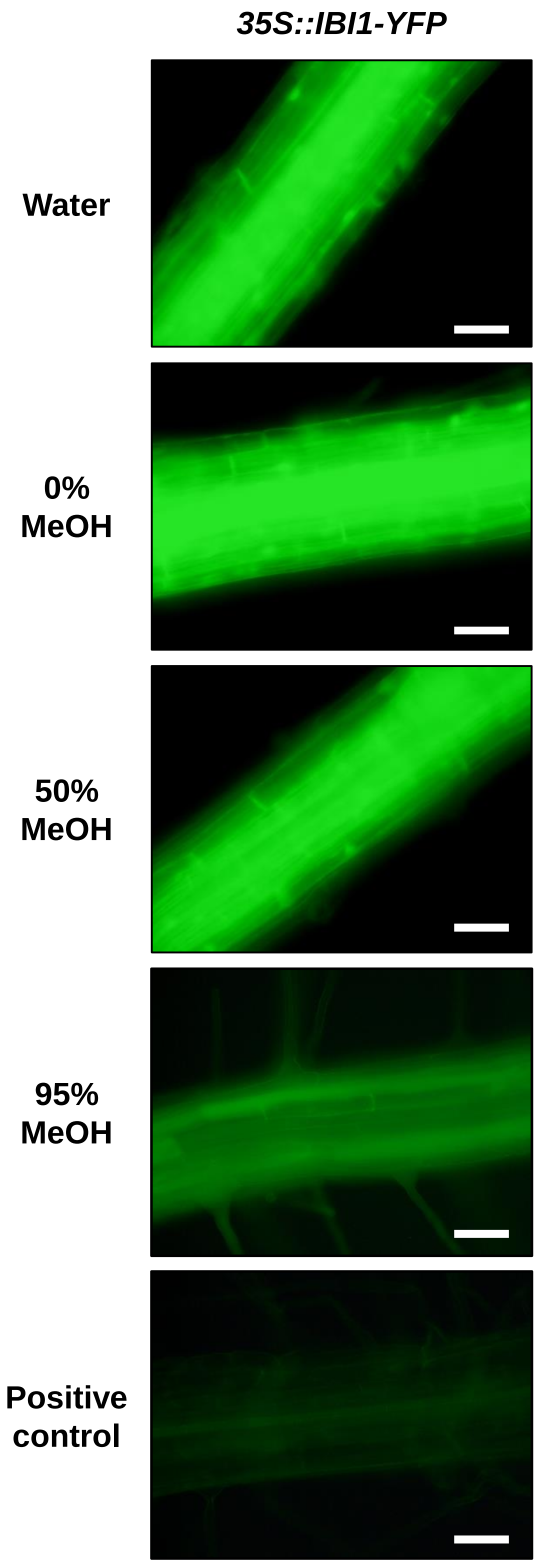

Supplemental Figure S4. Epi-fluorescence microscopy analysis of cell damage in Arabidopsis roots after exposure to MeOH-containing extraction solutions.

Transgenic roots producing the cytoplasmic aspartyl-tRNA synthase IBI1 fused to YFP (*35S::IBI1:YFP*; Luna *et al.*, 2014) were incubated for 1 min in water or acidified extraction solutions with increasing MeOH concentration (0, 50 or 95% MeOH, v/v + 0.05% formic acid, v/v). After incubation, roots were then rinsed in sterile water, and analysed for YFP fluorescence. Photographs show representative examples from observations of at least 12 roots for each treatment. As a positive control for cell damage, roots were incubated in 100% MeOH for 15 min. The experiment was performed four time with similar results. Scale bars: 50 µm.
