# Supplementary material for: Metabolite profiling of non‐sterile rhizosphere soil
Source: Plant J. 2017 Aug 31;92(1):147–62. doi: 10.1111/tpj.13639 (PMC5639361; doi:10.1111/tpj.13639)
Supplement: Supplementary file 5 — Figure S5. Reproducibility of metabolite profiles between experiments. [file TPJ-92-147-s005.pdf]

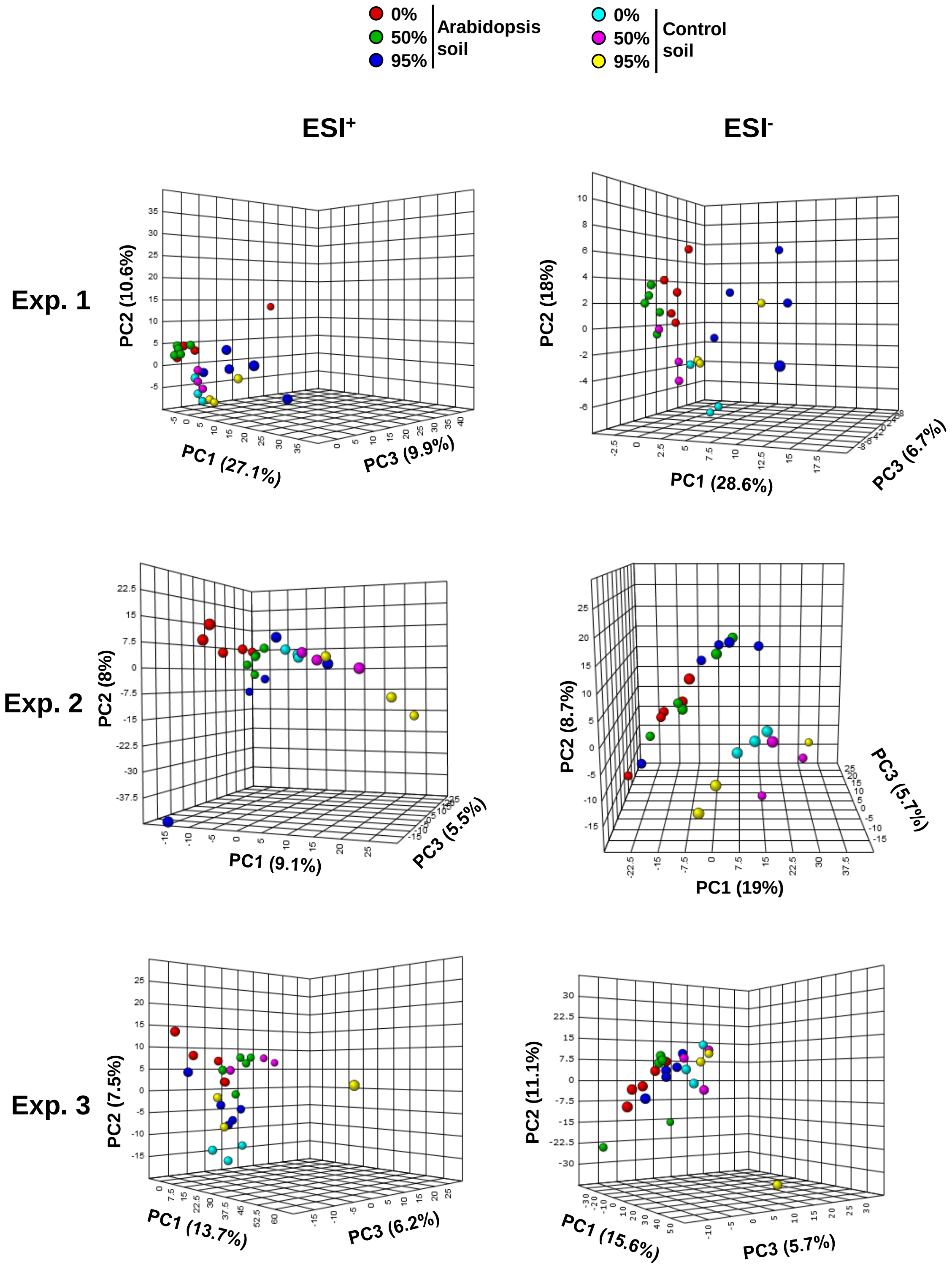

Supplemental Figure S5. Reproducibility of differences in metabolite profiles between control and Arabidopsis soil over three independent experiments.

Shown are unsupervised three-dimensional principal component analyses (3D-PCA) from extracts by the different solutions (indicated by % MeOH). Ions (*m/z* values) were obtained by UPLC-Q-TOF in positive (ESI$^+$, left panels) and negative (ESI$^-$, right panel) ionization modes. Analysis was carried out with MetaboAnalyst (v. 3.0), after median normalization, cube-root transformation and Pareto scaling of data. In parentheses are shown the percentages of variation explained by each principal component.
