# Supplementary material for: Metabolite profiling of non‐sterile rhizosphere soil
Source: Plant J. 2017 Aug 31;92(1):147–62. doi: 10.1111/tpj.13639 (PMC5639361; doi:10.1111/tpj.13639)
Supplement: Supplementary file 6 — Figure S6. Binary PLS‐DA analysis of metabolite profiles. [file TPJ-92-147-s006.pdf]

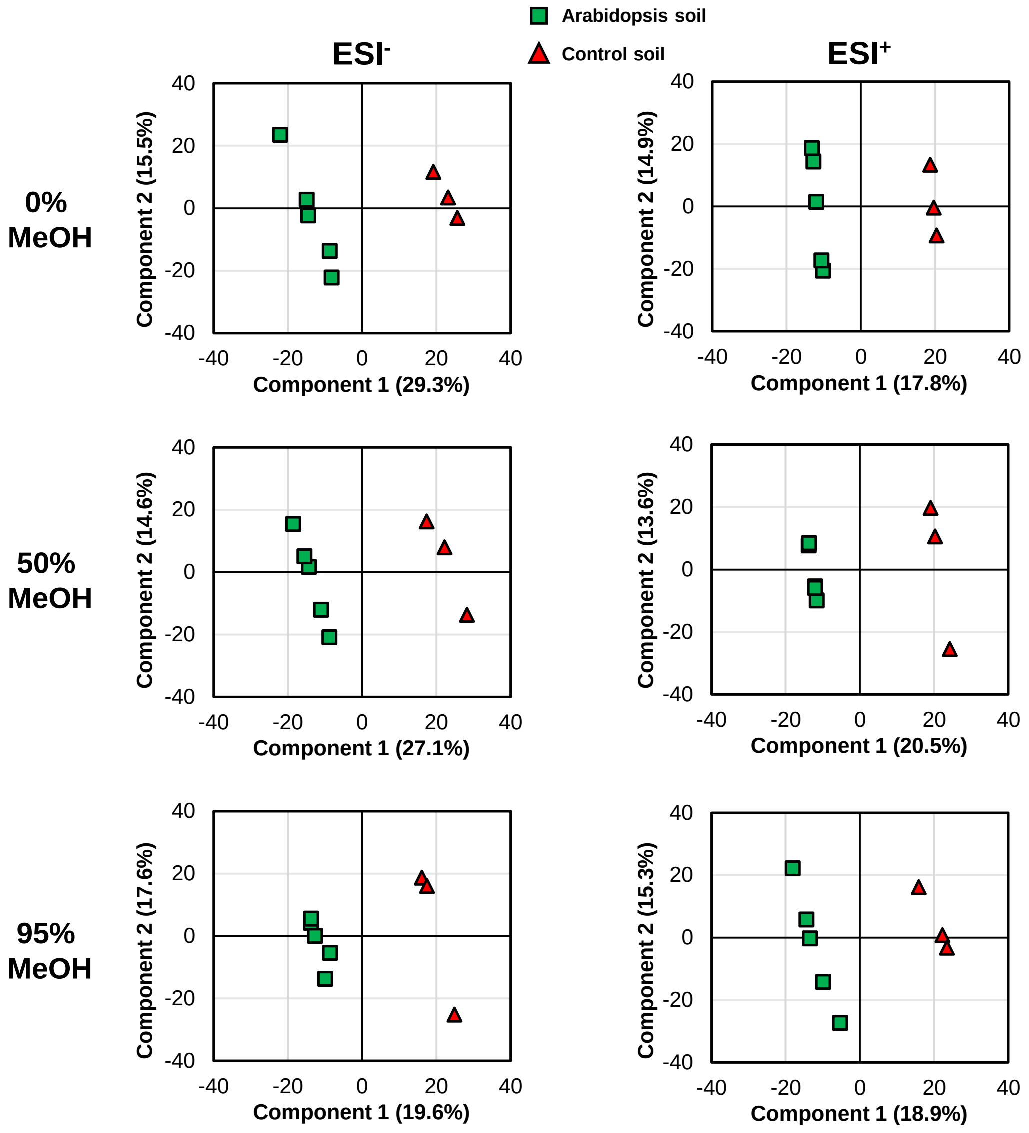

Supplemental Figure S6. Binary PLS-DA analysis of metabolite profiles from control soil and Arabidopsis soil for different extraction solutions (indicated by % MeOH).

Ions (*m/z* values) were obtained by UPLC-Q-TOF analysis in both positive (ESI^+, left panels) and negative (ESI^-, right panel) ionization mode. Prior to analysis, data were median-normalized, cube-root-transformed and Pareto-scaled. All R^2 (correlation) and Q^2 (predictability) values of PLS-DA models were above 0.94 and 0.59, respectively.
