# Supplementary material for: Metabolite profiling of non‐sterile rhizosphere soil
Source: Plant J. 2017 Aug 31;92(1):147–62. doi: 10.1111/tpj.13639 (PMC5639361; doi:10.1111/tpj.13639)
Supplement: Supplementary file 7 — Figure S7. Details of quantitative differences in metabolites. [file TPJ-92-147-s007.pdf]

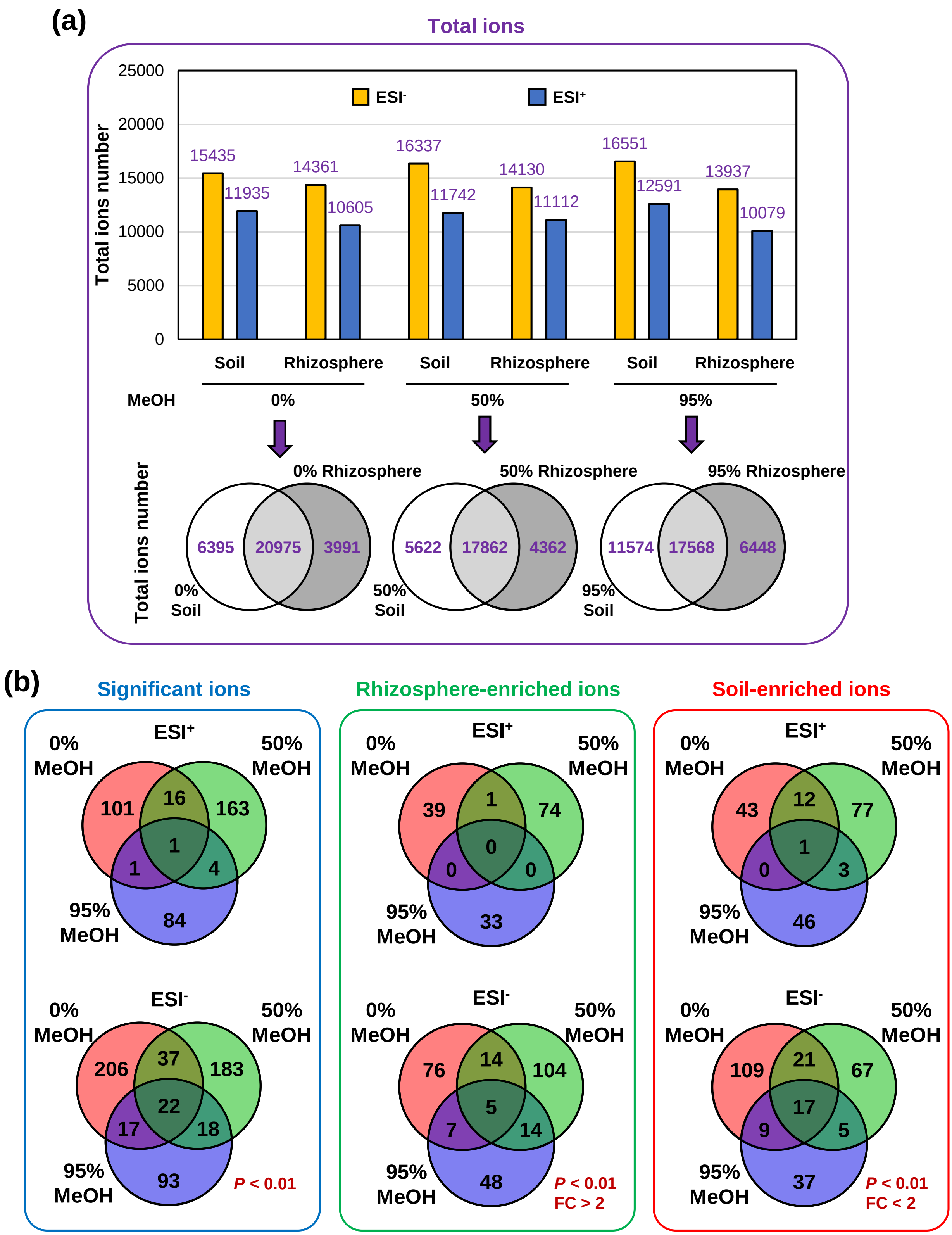

Supplemental Figure S7. Quantitative differences in detected ions (UPLC-Q-TOF) between extracts from control and Arabidopsis soil.

(**a**) Total numbers of ions (top) detected in Arabidopsis soil and control soil after extraction with the different solutions (indicated by % MeOH). Venn diagrams (bottom) show overlap in total ion numbers between extracts for each extraction solution.
(**b**) Venn diagrams showing overlap in cations (ESI^+) and anions (ESI^-) that are statistically different between control and Arabidopsis soil (left panel; $P < 0.01$, Welch's *t*-test), that are enriched in extracts from Arabidopsis soil (middle panel; > 2-fold enrichment to soil at $P < 0.01$, Welch's *t*-test), and that enriched are in extracts from control soil (right panel; < 2-fold enrichment to soil at $P < 0.01$, Welch's *t*-test).
