# Supplementary material for: Metabolite profiling of non‐sterile rhizosphere soil
Source: Plant J. 2017 Aug 31;92(1):147–62. doi: 10.1111/tpj.13639 (PMC5639361; doi:10.1111/tpj.13639)
Supplement: Supplementary file 8 — Figure S8. Relative quantities of benzoxazinoids. [file TPJ-92-147-s008.pdf]

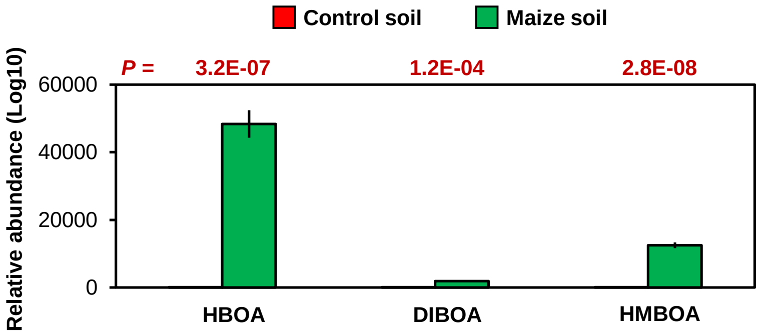


**Supplemental Figure S8.** Relative quantities of selected benzoxazinoid ions in extracts from maize soil and corresponding control soil.

Selective ions (*m/z*) of HBOA (2-hydroxy-4H-1,4-benzoxazin-3-one), DIBOA (2,4-dihydroxy-1,4-benzoxazin-3-one) and 2-hydroxy-7-methoxy-2H-1,4-benzoxazin-3(4H)-one were detected on the basis of retention time and *m/z* value, using UPLC-Q-TOF ($ESI^+$, Δppm = 0). Charts indicate means of relative abundances (*n* = 5, ± SEM). Levels of statistical significance are indicated in red above the corresponding bars (Student's *t*-test).
