# Supplementary material for: Metabolite profiling of non‐sterile rhizosphere soil
Source: Plant J. 2017 Aug 31;92(1):147–62. doi: 10.1111/tpj.13639 (PMC5639361; doi:10.1111/tpj.13639)
Supplement: Supplementary file 9 — Figure S9. Profiling distal rhizosphere chemistry. [file TPJ-92-147-s009.pdf]

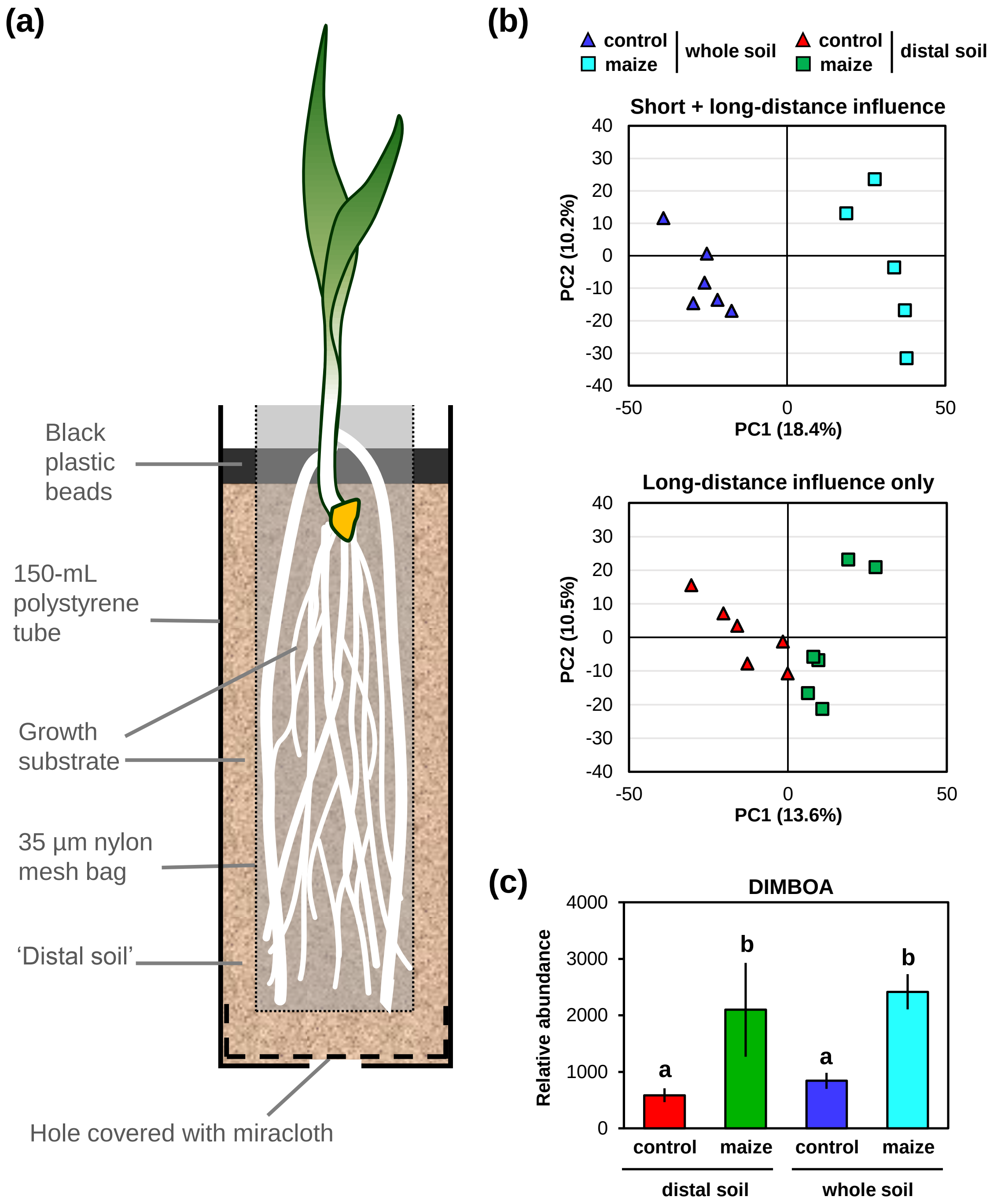

Supplemental Figure S9. Profiling distal rhizosphere chemistry.

(**a**) Experimental growth system to profile chemistry of distal rhizosphere fractions. Maize was grown within nylon mesh bags inside 150-mL tubes, containing agricultural soil from arable farmland and perlite (75:25, v/v). Similar plant-free tubes were constructed as controls. After 24 days of growth, chemicals were extracted with the acidified 50% MeOH solution from either the entire pot (whole soil), or the soil surrounding the root containing mesh bag after its careful removal (distal soil).
(**b**) Binary PCAs showing chemical rhizosphere effects in whole soil fractions (upper panel; short + long distance influence) and distal soil fractions (lower panel; long distance influence), illustrating that the rhizosphere extends beyond soil that is closely associated with roots.
(**c**) Targeted quantification of DIMBOA by UPLC-Q-TOF. Shown are average ion intensities (± SEM; *n* = 6), normalised by soil weight. Letters indicate statistically significant differences between soil types (Student's *t*-test, $P < 0.05$).
