# Supplementary material for: Metabolite profiling of non‐sterile rhizosphere soil
Source: Plant J. 2017 Aug 31;92(1):147–62. doi: 10.1111/tpj.13639 (PMC5639361; doi:10.1111/tpj.13639)
Supplement: Supplementary file 10 — Table S1. Putative identification of Arabidopsis metabolic markers. [file TPJ-92-147-s010.pdf]

Supplemental Table S1. Putative identification of Arabidopsis rhizosphere- and control soil-enriched metabolic markers

[1] Percentages indicate relative MeOH contents of the extraction solutions.

[2] *P* values are derived from ANOVA followed by false discovery rate correction (Benjamini-Hochberg).

[3] Retention times (RT) and accurate *m/z* values, detected by UPLC-Q-TOF in negative (-) or positive (+) ion mode.

[4] Predicted parameters from the METLIN database using the detected accurate *m/z.*

[5] Putative metabolites and their corresponding pathways were validated by information from the PubMed chemical database.

[6] Putative metabolites that unlikely accumulate in (rhizosphere) soil.

RHIZOSPHERE -ENRICHED

Sample	Extraction solution[1]	*P* value[2]	RT (min)[3]	Detected *m/z* [3]	Ion mode [3]	Predicted mass[4]	*Adduct* [4]	Δppm[4]	Putative Compound[4]	Predicted Formula[4]	Putative Pathway[5]	soil- or plant-derived[6]
	0%	5.9E-05	7.4	336.117	-	337.131	[M-H]-	21	(S)-cis-N-methylstylopine	C20H19NO4	Alkaloids	
	95%	8.2E-03	1.1	612.166	-	567.189	[M+FA-H]-	35	Neoacrimarine H	C33H29NO8	Alkaloids	
	0%	3.1E-03	5.2	657.315	+	656.310	[M+H]+	3	Disinomenine	C38H44N2O8	Alkaloids	
	0%	1.8E-05	5.9	116.052	+	151.063	[M+H-2H2O]+	12	2-Phenylglycine	C8H9NO2	Amino acids	
	95%	1.2E-03	5.0	265.106	+	264.104	[M+H]+	21	Thiamine	C12H16N4OS	Amino acids	
	0%	7.5E-03	6.9	109.039	-	110.048	[M-H]-	15	Imidazole-4-acetaldehyde	C5H6N2O	Amino acids	
	50%	9.5E-04	7.2	414.322	+	413.314	[M+H]+	1	N-stearoyl glutamic acid	C23H43NO5	Amino acids	
	95%	2.4E-03	1.3	668.121	-	669.144	[M-H]-	23	Enterochelin	C30H27N3O15	Amino acids	
	50%	3.1E-04	1.4	580.656	-	599.686	[M-H2O-H]-	19	Calcium trimetaphosphate	Ca3H6O18P6	Calcium source from Plants	
	50%	6.5E-05	1.2	620.662	-	599.686	[M+Na-2H]-	2	Calcium trimetaphosphate	Ca3H6O18P6	Calcium source from Plants	
	0%	2.6E-03	9.3	776.177	+	775.195	[M+H]+	32	Reduced coenzyme F420	C29H38N5O18P	Carbohydrates	
	50, 95%	6.0E-04	7.3	383.156	-	384.163	[M-H]-	0	2,3-Butanediol apiosylglucoside	C15H28O11	Flavonoids	
	50%	5.2E-05	1.3	699.141	-	654.143	[M+FA-H]-	0	Tamarixetin 5-glucoside-7-glucuronide	C28H30O18	Flavonoids	
	50%	8.1E-04	1.2	727.147	-	728.180	[M-H]-	35	Primflaside	C31H36O20	Flavonoids	
	0%	9.5E-04	9.0	479.275	+	478.272	[M+H]+	8	3-Geranyl-4,2\',4\',6\'-tetrahydroxy-5-prenyldihydrochalcone	C30H38O5	Flavonoids	Unlikely
	0%	7.1E-03	1.2	648.153	-	649.177	[M-H]-	25	Pyranodelphinin A	C30H33O16	Flavonoids	
	50%	1.9E-04	1.2	665.136	-	666.143	[M-H]-	0	Cyanidin 3-(6\'\'-malonylsambubioside)	C29H30O18	Flavonoids	
	0%	4.3E-03	9.4	452.924	-	453.942	[M-H]-	24	4,4'-Diisothiocyano-2,2'-stilbenedisulfonic acid	C16H10N2O6S4	Isothiocyanates	Unlikely
	0%	2.1E-06	8.1	347.327	+	364.334	[M+H-H2O]+	12	5,9-tetracosadienoic acid	C24H44O2	Lipids	
	0%	4.8E-03	6.7	594.585	+	593.575	[M+H]+	5	Ceramide	C38H75NO3	Lipids	
	0%	4.4E-03	9.1	664.458	+	663.448	[M+H]+	4	Phosphoserine	C34H66NO9P	Lipids	
	50%	6.0E-07	1.3	816.548	-	817.562	[M-H]-	8	Phosphatidylethanolamine	C47H80NO8P	Lipids	
	50%	3.7E-03	7.3	395.346	+	394.324	[M+H]+	38	Dehydroergosterol	C28H42O	Lipids	
	50%	3.1E-03	6.4	420.293	+	437.291	[M+H-H2O]+	12	Phosphatidylethanolamine	C21H44NO6P	Lipids	
	95%	2.0E-05	7.7	441.298	+	440.290	[M+H]+	1	26,26,26-trifluoro-25-hydroxy-27-norcholecalciferol	C26H39F3O2	Lipids	Unlikely
	50%	6.1E-04	1.6	480.787	-	499.803	[M-H2O-H]-	4	Butter acids	C15H8Cl8O2	Lipids	
	50%	1.6E-03	7.2	485.382	+	520.392	[M+H-2H2O]+	6	Ginsenoyne A linoleate	C35H52O3	Lipids	
	50%	3.5E-03	9.1	503.461	+	538.460	[M+H-2H2O]+	27	Diacylglycerol	C33H62O5	Lipids	
	50%	3.5E-04	9.3	658.604	+	675.593	[M+H-H2O]+	21	Diacylglycerol	C43H79D5O5	Lipids	
	95%	3.8E-03	1.1	672.565	-	673.549	[M-H]-	34	Glucosylceramide	C38H75NO8	Lipids	
	50%	8.7E-04	9.2	763.519	+	762.520	[M+H]+	10	Phosphatidic acid	C44H75O8P	Lipids	
	0%	3.8E-06	9.0	771.658	-	772.658	[M-H]-	9	Triacylglycerol	C49H88O6	Lipids	
	95%	1.4E-03	2.8	809.600	-	810.614	[M-H]-	8	Phosphatidic acid	C47H87O8P	Lipids	
	95%	6.0E-07	1.3	816.548	-	817.562	[M-H]-	8	Phosphatidylethanolamine	C47H80NO8P	Lipids	
	0%	3.1E-03	1.4	834.524	-	835.536	[M-H]-	6	Phosphoserine	C46H78NO10P	Lipids	
	50%	8.2E-03	1.2	856.508	-	857.521	[M-H]-	6	Phosphoserine	C48H76NO10P	Lipids	
	95%	5.0E-04	6.7	1023.795	+	1022.830	[M+H]+	41	Triacylglycerol	C68H110O6	Lipids	
**Rhizosphere**	95%	1.9E-03	1.5	1057.815	-	1012.846	[M+FA-H]-	27	Triacylglycerol	C67H112O6	Lipids	
	95%	< E-09	6.3	265.182	+	264.173	[M+H]+	8	12-Oxo-2,3-dinor-10,15-phytodienoic acid	C16H24O3	Lipids/JA metabolism	
	0%	7.8E-04	1.7	92.927	-	111.948	[M-H2O-H]-	30	2,2-Dichloroacetaldehyde	C2H2Cl2O	Miscellaneous	Unlikely
	0%	4.8E-03	1.2	306.918	+	305.910	[M+H]+	1	Mitobronitol	C6H12Br2O4	Miscellaneous/Alcohol	Unlikely
	0%	2.0E-03	1.1	338.812	-	357.844	[M-H2O-H]-	41	2,2\',4,4\',5,5\'-Hexachlorobiphenyl	C12H4Cl6	Miscellaneous/Aromatics	Unlikely
	0, 50, 95%	1.0E-06	1.0	358.836	-	359.837	[M-H]-	16	Haloprogin	C9H4Cl3IO	Miscellaneous/Aromatics	
	50%	2.8E-03	1.1	380.818	-	359.837	[M+Na-2H]-	15	Haloprogin	C9H4Cl3IO	Miscellaneous/Aromatics	
	50%	5.4E-06	1.5	538.675	-	493.689	[M+FA-H]-	21	Decachlorobiphenyl	C12Cl10	Miscellaneous/Aromatics	Unlikely
	50%	< E-09	1.9	203.025	+	202.011	[M+H]+	31	4-carboxy-2-hydroxy-cis,cis-muconic acid	C7H6O7	Miscellaneous/Carboxylic acids	Unlikely
	0, 50%	1.9E-03	1.1	216.907	-	235.932	[M-H2O-H]-	30	2-Bromomaleylacetate	C6H5BrO5	Miscellaneous/Carboxylic acids	Unlikely
	0%	1.3E-04	1.4	318.838	-	337.863	[M-H2O-H]-	19	1,2,3,7,8-Pentachlorodibenzofuran	C12H3Cl5O	Miscellaneous/Dioxins	Unlikely
	95%	7.1E-03	1.2	680.140	-	635.142	[M+FA-H]-	0	Aflatoxin B1 exo-8,9-epoxide-GSH	C27H29N3O13S	Miscellaneous/Mycotoxins	Unlikely
	95%	1.1E-05	4.6	1051.437	-	1006.436	[M+FA-H]-	2	Oxytocin	C43H66N12O12S2	Miscellaneous/Peptide hormones	Unlikely
	0%	3.2E-03	6.8	1126.544	+	1125.501	[M+H]+	31	[Tyr(PO3H2)4]-Angiotensin II	C50H72N13O15P	Miscellaneous/Peptide hormones	Unlikely
	0%	6.9E-05	2.0	181.994	+	217.006	[M+H-2H2O]+	3	PROPANIL	C9H9Cl2NO	Miscellaneous/Pesticides	Unlikely
	0%	7.8E-03	4.7	291.058	-	270.089	[M+Na-2H]-	20	4-O-Methylpinosylvic acid	C16H14O4	Phenylpropanoids	
	95%	4.3E-03	1.2	646.088	-	665.125	[M-H2O-H]-	28	NADH	C21H29N7O14P2	Pyridine nucleotides	
	50%	8.0E-05	5.8	360.978	+	395.986	[M+H-2H2O]+	14	Sesquiterpene	C15H23Br2Cl	Terpenoids	Unlikely
	95%	3.7E-03	9.2	74.781	-						Unknown	
	0%	6.7E-05	9.3	87.183	+						Unknown	
	50%	7.8E-03	1.4	218.880	-						Unknown	
	50%	1.5E-05	9.3	225.793	+						Unknown	
	0%	8.6E-04	1.2	262.896	-						Unknown	
	50%	3.3E-03	1.8	279.966	+						Unknown	
	0, 50, 95%	1.8E-09	0.9	396.789	-						Unknown	
	95%	5.2E-03	1.7	400.792	-						Unknown	
	95%	2.6E-05	6.0	430.389	+						Unknown	
	0%	4.1E-05	4.5	444.801	-						Unknown	
	50%	2.9E-05	1.3	470.700	-						Unknown	
	50, 95%	2.2E-04	1.6	518.751	-						Unknown	
	50%	9.0E-03	7.2	540.856	+						Unknown	
	50%	1.6E-03	1.1	558.693	-						Unknown	
	95%	5.8E-03	7.6	598.899	+						Unknown	
	50%	2.7E-06	1.2	618.651	-						Unknown	
	0%	9.5E-03	2.8	620.761	-						Unknown	
	0%	6.6E-03	9.4	766.946	+						Unknown	
	95%	1.7E-04	7.1	862.800	+						Unknown	
	95%	8.4E-07	9.3	1067.185	+						Unknown	
	95%	5.5E-04	9.3	1119.101	+						Unknown	

SOIL-ENRICHED

Sample	Extraction solution[1]	*P* value[2]	RT (min)[3]	Detected *m/z*[3]	Ion mode	Predicted mass[4]	*Adduct*[4]	Δppm[4]	Putative Compound[4]	Predicted Formula[4]	Putative Pathway[5]	soil- or plant-derived[6]
	50%	< E-13	1.2	130.086	-	131.095	*[M-H]-*	10	L-Isoleucine	C6H13NO2	Amino acids	
	50%	1.89E-04	1.0	216.035	+	215.019	*[M+H]+*	38	O-Phospho-4-hydroxy-L-threonine	C4H10NO7P	Amino acids	
	50%	5.77E-05	3.0	245.079	+	280.092	*[M+H-2H2O]+*	0	Methionyl-Methionine	C10H20N2O3S2	Amino acids	
	50%	1.12E-04	1.4	284.061	+	283.046	*[M+H]+*	28	N2-Acetyl-L-aminoadipyl-δ-phosphate	C8H14NO8P	Amino acids	
	50%	1.41E-07	1.3	1103.693	+	215.986	*[M+H]+*	46	S-Methyl-3-phospho-1-thio-D-glycerate	C4H9O6PS	Carbohydrates	
	95%	8.67E-05	6.1	335.033	+	334.032	*[M+H]+*	20	Heptahydroxyflavone	C15H10O9	Flavonoids	
	95%	2.36E-03	7.6	553.287	+	570.289	*[M+H-H2O]+*	1	2-O-(beta-D-galactopyranosyl-(1->6)-beta-D-galactopyranosyl) 2S,3R-dihydroxytridecanoic acid	C25H46O14	Lipids	Unlikely
	95%	6.51E-08	8.0	553.513	+	552.491	*[M+H]+*	27	Linoleyl arachidonate	C38H64O2	Lipids	
	50%	2.05E-05	4.9	620.535	+	619.530	*[M+H]+*	3	Diacylglycerol	C39H71D5O5	Lipids	
	0%	5.33E-03	9.4	701.408	+	700.432	*[M+H]+*	43	Phosphatidylglycerol	C37H65O10P	Lipids	
	95%	5.36E-03	8.1	172.063	-	173.069	*[M-H]-*	8	2,6-Piperidinedicarboxylic acid	C7H11NO4	Miscellaneous	
	0, 50%	6.49E-06	0.8	184.926	-	185.932	*[M-H]-*	8	4-Bromo-3,5-cyclohexadiene-1,2-dione	C6H3BrO2	Miscellaneous	Unlikely
	0%	3.48E-03	7.9	296.181	+	331.194	*[M+H-2H2O]+*	0	Currayanine	C23H25NO	Miscellaneous	Unlikely
	50%	2.64E-03	9.4	495.013	+	494.019	*[M+H]+*	27	Sodium cumeneazo-β-naphthol disulfonate	C19H16N2Na2O7S2	Miscellaneous	Unlikely
	0%	7.92E-07	9.3	163.943	+	198.948	*[M+H-2H2O]+*	47	Bronopol	C3H6BrNO4	Miscellaneous/Antimicrobials	Unlikely
	95%	3.16E-03	9.4	1088.122	+	1123.158	*[M+H-2H2O]+*	21	Mycolic acid	C77H150O3	Miscellaneous/Antimicrobials	Unlikely
	50%	1.01E-05	0.8	110.009	+	145.020	*[M+H-2H2O]+*	18	3,4-Dehydrothiomorpholine-3-carboxylate	C5H7NO2S	Miscellaneous/Carboxylic acids	
	0, 95%	5.65E-13	1.1	148.043	+	147.035	*[M+H]+*	2	Thiomorpholine 3-carboxylate	C5H9NO2S	Miscellaneous/Carboxylic acids	
	0, 50%	7.86E-06	1.1	346.099	+	345.091	*[M+H]+*	0	Clopamide	C14H20ClN3O3S	Miscellaneous/Diuretics	Unlikely
	50%	3.85E-03	1.7	346.096	+	345.091	*[M+H]+*	7	Clopamide	C14H20ClN3O3S	Miscellaneous/Diuretics	Unlikely
	50%	5.41E-03	6.9	382.128	-	383.134	*[M-H]-*	2	Fluazifop butyl	C19H20F3NO4	Miscellaneous/Herbicides	Unlikely
	95%	1.44E-03	2.9	303.049	-	304.058	*[M-H]-*	4	Brompheniramine (monodemethylated)	C15H17BrN2	Miscellaneous/Histamines	Unlikely
	0, 50%	1.44E-04	0.8	183.928	-	184.934	*[M-H]-*	8	Iodoacetamide	ICH2CONH2	Miscellaneous/Lipids	Unlikely
	95%	1.67E-04	8.6	584.329	-	585.345	*[M-H]-*	15	Janthitrem B	C37H47NO5	Miscellaneous/Mycotoxins	Unlikely
	0%	8.26E-03	1.2	234.048	+	269.061	*[M+H-2H2O]+*	0	2-(p-Methoxyphenyl)-3-(m-chlorophenyl)acrylonitrile	C16H12ClNO	Miscellaneous/Nitriles	Unlikely
	50%	1.63E-05	0.8	199.045	+	198.039	*[M+H]+*	5	Nitrofurazone	C6H6N4O4	Miscellaneous/Nitrofurans	Unlikely
	95%	< E-13	1.7	288.046	-	289.054	*[M-H]-*	1	Isocarbophos	C11H16NO4PS	Miscellaneous/Pesticides	Unlikely
	50%	4.74E-04	1.4	303.871	-	258.876	*[M+FA-H]-*	10	Tecnazene	C6HCl4NO2	Miscellaneous/Pesticides	Unlikely
	95%	2.65E-04	7.1	775.262	+	774.247	*[M+H]+*	9	7,8-Dihydromethanopterin	C30H43N6O16P	Miscellaneous/Pteridines	
	0%	5.61E-03	7.7	549.916	+	584.937	*[M+H-2H2O]+*	14	HLo7	C15H17I2N5O4	Miscellaneous/Pyridines	
	95%	1.64E-04	4.2	596.085	-	551.106	*[M+FA-H]-*	32	11-O-Demethylpradimicinone II	C27H21NO12	Miscellaneous/Quinones	Unlikely
	0%	1.88E-03	9.3	405.185	+	404.175	*[M+H]+*	5	Chlormadinone acetate	C23H29ClO4	Miscellaneous/Steroids	Unlikely
	0%	5.20E-03	7.8	457.263	+	456.255	*[M+H]+*	2	Lithocholic acid sulfate	C24H40O6S	Miscellaneous/Steroids	Unlikely
	0%	1.66E-04	1.1	370.066	+	369.059	*[M+H]+*	2	4-Pyridinol, 2-[[[5-(difluoromethoxy)-1H-benzimidazol-2-yl]sulfinyl]methyl]-3-methoxy-	C15H13F2N3O4S	Miscellaneous/Sulfoxides	Unlikely
	0, 50, 95%	2.37E-06	0.9	210.949	-	211.960	*[M-H]-*	18	Thiotropocin	C8H4O3S2	Miscellaneous/Tropolones	Unlikely
	0, 50%	1.00E-04	1.3	216.983	+	215.986	*[M+H]+*	46	S-Methyl-3-phospho-1-thio-D-glycerate	C4H9O6PS	Organic acids	
	50%	2.61E-04	3.3	263.094	+	262.089	*[M+H]+*	8	Thiamine aldehyde	C12H14N4OS	Organic acids	
**Control soil**	0, 50%	2.26E-07	0.8	182.929	-	183.935	*[M-H]-*	5	Arsonoacetate	C2H5AsO5	Organoarsonic acids	Unlikely
	0%	4.39E-04	6.5	321.094	+	320.090	*[M+H]+*	8	4-Coumaroylshikimate	C16H16O7	Phenylpropanoids	
	0%	6.17E-07	9.4	1049.027	+	1084.067	*[M+H-2H2O]+*	25	Punicalagin	C48H28O30	Phenylpropanoids	Unlikely
	50%	6.61E-03	2.2	1117.153	-	1072.183	*[M+FA-H]-*	25	CoA-glutathione	C31H51N10O22P3S2	Purines	
	50%	1.50E-08	9.3	349.184	+	348.178	*[M+H]+*	4	cis-10-Hydroxylinalyl oxide 7-glucoside	C16H28O8	Terpenoids	
	0, 50%	1.48E-07	0.9	61.987	-						Unknown	
	0, 50%	2.61E-05	1.6	61.988	-						Unknown	
	0, 50%	1.78E-05	1.0	123.941	+						Unknown	
	95%	3.03E-05	1.1	160.841	-						Unknown	
	0%	3.15E-04	1.9	165.936	-						Unknown	
	95%	5.05E-04	2.0	175.968	-						Unknown	
	0, 95%	7.65E-03	0.8	192.957	-						Unknown	
	95%	3.88E-06	4.8	195.925	+						Unknown	
	0, 50%	4.24E-03	1.8	209.948	-						Unknown	
	0, 50%	5.78E-06	0.9	211.946	-						Unknown	
	95%	7.37E-04	9.7	245.894	-						Unknown	
	95%	4.85E-04	1.3	261.870	-						Unknown	
	95%	7.08E-04	1.3	299.842	-						Unknown	
	95%	3.80E-03	5.4	307.150	+						Unknown	
	95%	5.18E-03	4.9	319.154	+						Unknown	
	0%	1.00E-04	1.7	325.894	-						Unknown	
	0%	3.88E-03	1.3	351.873	-						Unknown	
	95%	1.72E-04	1.4	379.788	-						Unknown	
	0%	1.40E-04	1.4	409.834	-						Unknown	
	0, 50%	1.80E-05	1.3	414.120	+						Unknown	
	95%	3.50E-03	1.5	443.807	-						Unknown	
	50%	8.21E-06	1.1	445.806	-						Unknown	
	50%	1.70E-07	1.5	475.778	-						Unknown	
	95%	3.03E-05	9.4	741.928	-						Unknown	
	95%	5.54E-03	8.5	776.674	+						Unknown	
	95%	1.40E-04	6.7	891.384	+						Unknown	
	0%	3.83E-03	2.2	992.858	-						Unknown	
	0%	3.95E-05	0.9	146.965	-						Unknown	
	0, 50%	9.75E-07	1.8	146.965	-						Unknown	
	0, 50%	1.72E-03	0.9	148.968	-						Unknown	
	0, 50%	1.16E-05	0.8	209.948	-						Unknown	
	0, 50%	9.40E-03	1.8	210.949	-						Unknown	
	0, 50%	3.24E-04	1.8	211.946	-						Unknown	
