# Supplementary material for: Metabolite profiling of non‐sterile rhizosphere soil
Source: Plant J. 2017 Aug 31;92(1):147–62. doi: 10.1111/tpj.13639 (PMC5639361; doi:10.1111/tpj.13639)
Supplement: Supplementary file 11 — Table S2. Putative identification of maize metabolic markers. [file TPJ-92-147-s011.pdf]

Supplemental Table S2. Putative identification of maize rhizosphere- and control soil-enriched metabolic markers

[1] Fold-change between maize rhizosphere samples and control soil samples.

[2] *P* values are derived from Welch's *t*-test.

[3] Retention times (RT) and accurate *m/z* values, detected by UPLC-Q-TOF in negative (-) or positive (+) ion mode.

[4] Predicted parameters from the METLIN database using the detected accurate *m/z.*

[5] Putative metabolites and their corresponding pathways were validated by information from the PubMed chemical database.

[6] Putative metabolites that unlikely accumulate in (rhizosphere) soil.

RHIZOSPHERE -ENRICHED

Sample	FC	*P* value[2]	RT (min)[3]	Detected *m/z*[3]	Ion mode[3]	Predicted mass[4]	*Adduct*[4]	Δppm[4]	Putative Compound[4]	Predicted Formula[4]	Putative Pathway[5]	Soil- or plant-derived[6]
Rhizosphere	96	2.2E-05	2.8	656.303	-	675.325	*[M-H2O-H]-*	6	Jesaconitine	C35H49NO12	Alkaloids	Unlikely
	23	7.6E-03	3.8	512.139	-	513.142	*[M-H]-*	8	Dioxinoacrimarine A	C29H23NO8	Alkaloids	Unlikely
	16	2.6E-04	2.0	434.119	-	413.147	*[M+Na-2H]-*	7	Noscapine	C22H23NO7	Alkaloids	
	833	8.3E-06	2.0	410.106	+	387.117	*[M+Na]+*	0	HDMBOA-Glc	C16H21NO10	Benzoxazinoids	
	392	3.2E-07	2.2	166.050	+	165.043	*[M+H]+*	0	HBOA	C8H7NO3	Benzoxazinoids	
	298	1.8E-04	1.5	426.101	+	403.111	*[M+Na]+*	0	DIM2BOA-Glc	C16H21NO11	Benzoxazinoids	
	292	2.8E-08	2.1	196.061	+	195.053	*[M+H]+*	2	2-Hydroxy-7-methoxy-2H-1,4-benzoxazin-3(4H)-one	C9H9NO4	Benzoxazinoids	
	131	2.7E-06	1.5	410.106	+	387.117	*[M+Na]+*	0	HDMBOA-Glc	C16H21NO10	Benzoxazinoids	
	125	2.2E-05	1.5	380.095	+	357.106	*[M+Na]+*	0	HMBOA-Glc	C15H19NO9	Benzoxazinoids	
	92	5.4E-06	2.2	342.078	+	359.085	*[M+H-H2O]+*	13	TRIBOA-glucoside	C14H17NO10	Benzoxazinoids	
	59	9.7E-05	1.5	396.090	+	373.101	*[M+Na]+*	0	DIMBOA-Glc	C15H19NO10	Benzoxazinoids	
	55	8.5E-06	2.2	188.032	+	165.043	*[M+Na]+*	1	HBOA	C8H7NO3	Benzoxazinoids	
	712	3.0E-06	2.2	164.035	-	165.043	*[M-H]-*	1	HBOA	C8H7NO3	Benzoxazinoids	
	140	8.7E-05	2.0	424.085	-	403.111	*[M+Na-2H]-*	2	DIM2BOA-Glc	C16H21NO11	Benzoxazinoids	
	139	5.5E-05	1.5	402.104	-	403.111	*[M-H]-*	1	DIM2BOA-Glc	C16H21NO11	Benzoxazinoids	
	130	1.8E-04	1.5	356.099	-	357.106	*[M-H]-*	1	HMBOA-Glc	C15H19NO9	Benzoxazinoids	
	92	4.3E-05	1.5	386.109	-	387.117	*[M-H]-*	0	HDMBOA-Glc	C16H21NO10	Benzoxazinoids	
	44	1.4E-04	1.5	372.094	-	373.101	*[M-H]-*	1	DIMBOA-Glc	C15H19NO10	Benzoxazinoids	
	41	3.0E-03	1.6	224.054	-	225.064	*[M-H]-*	10	2-Hydroxy-4,7-dimethoxy-2H-1,4-benzoxazin-3(4H)-one	C10H11NO5	Benzoxazinoids	
	34	2.5E-04	2.0	356.099	-	357.106	*[M-H]-*	0	HMBOA-Glc	C15H19NO9	Benzoxazinoids	
	34	6.3E-05	2.0	386.109	-	387.117	*[M-H]-*	0	HDMBOA-Glc	C16H21NO10	Benzoxazinoids	
	13	1.2E-04	1.5	462.060	-	181.038	*[M-H]-*	22	DIBOA	C8H7NO4	Benzoxazinoids	
	104	8.7E-03	2.0	622.675	+	599.686	*[M+Na]+*	0	Calcium trimetaphosphate	Ca3H6O18P6	Calcium source from Plants	
	64	1.2E-05	2.0	452.097	-	431.121	*[M+Na-2H]-*	3	Ribosylzeatin phosphate	C15H22N5O8P	Cytokinins	
	27	1.5E-03	2.2	398.110	-	377.134	*[M+Na-2H]-*	5	Kinetin-7-N-glucoside	C16H19N5O6	Cytokinins	
	607	2.2E-05	2.0	857.239	+	874.238	*[M+H-H2O]+*	4	Quercetin 3-xylosyl-(1->3)-rhanosyl-(1->6)-[apiosyl-(1->2)-galactoside]	C37H46O24	Flavonoids	
	298	2.7E-05	2.0	828.233	+	863.225	*[M+H-2H2O]+*	25	Cyanidin 3-O-[b-D-Xylopyranosyl-(1->2)-[(4-hydroxybenzoyl)-(->6)-b-D-glucopyranosyl-(1->6)]-b-D-galactopyranoside]	C39H43O22	Flavonoids	
	288	1.0E-05	2.0	379.089	+	356.090	*[M+Na]+*	26	6,7,3\'-Trimethoxy-4\',5\'-methylenedioxyisoflavone	C19H16O7	Flavonoids	
	179	6.2E-03	1.9	824.197	+	801.209	*[M+Na]+*	1	Delphinidin 3-lathyroside 5-(6-acetylglucoside)	C34H41O22	Flavonoids	
	151	3.2E-04	1.9	615.661	+	1185.330	*[M+2Na]2+*	11	Cyanidin 3-(disinapoylsophoroside) 5-glucoside	C55H61O29	Flavonoids	
	129	9.4E-04	1.5	799.186	+	798.164	*[M+H]+*	17	Apigenin 4\'-(2\'\'-feruloylglucuronosyl)-(1->2)-glucuronide	C37H34O20	Flavonoids	
	97	4.3E-04	3.8	565.040	+	542.037	*[M+Na]+*	25	8-Hydroxyapigenin 8-(2\'\'-sulfatoglucuronide)	C21H18O15S	Flavonoids	
	95	7.4E-03	2.0	858.241	+	816.211	*[M+ACN+H]+*	4	Kaempferol 3-(6\'\'-sinapylglucosyl)-(1->2)-galactoside	C38H40O20	Flavonoids	
	84	1.5E-03	2.0	859.168	+	858.170	*[M+H]+*	11	Delphinidin 3-(6\'\'-O-4-malyl-glucoside)-5-(6\'\'\'-O-1-malyl-glucoside)	C35H38O25	Flavonoids	
	81	2.8E-06	2.0	441.120	+	476.132	*[M+H-2H2O]+*	2	Hesperetin 7-O-glucuronide	C23H24O11	Flavonoids	
	60	2.4E-04	2.2	357.584	+	713.157	*[M+2H]2+*	4	Delphinidin 3-(6''-malonyl glucoside) 5-glycoside	C30H33O20	Flavonoids	
	167	2.4E-05	2.2	276.551	+	507.114	*[M+2Na]2+*	17	Delphinidin 3-(acetylglucoside)	C23H23O13	Flavonoids	
	456	9.7E-05	2.0	432.115	-	433.113	*[M-H]-*	20	Pelargonidin 3-galactoside	C21H21O10	Flavonoids	
	110	6.5E-06	2.0	462.126	-	463.124	*[M-H]-*	20	Malvidin-3-O-xyloside	C22H23O11	Flavonoids	
	68	1.7E-04	2.0	433.118	-	434.121	*[M-H]-*	9	Naringenin-7-O-Glucoside	C21H22O10	Flavonoids	
	49	4.3E-05	1.5	440.079	-	419.098	*[M+Na-2H]-*	15	Cyanidin 3-arabinoside	C20H19O10	Flavonoids	
	36	6.9E-03	1.6	594.168	-	595.166	*[M-H]-*	15	Cyanidin 3-rhamnoside 5-glucoside	C27H31O15	Flavonoids	
	32	4.2E-05	1.5	470.091	-	449.108	*[M+Na-2H]-*	17	Cyanidin 3-galactoside	C21H21O11	Flavonoids	
	28	6.4E-05	2.0	463.126	-	464.132	*[M-H]-*	2	Hesperetin 7-O-glucoside	C22H24O11	Flavonoids	
	22	2.2E-04	3.0	176.907	-	433.113	*[M+Na-2H]-*	11	Petunidin 3-arabinoside	C21H21O10	Flavonoids	
	19	3.9E-05	2.0	454.093	-	433.113	*[M+Na-2H]-*	11	Petunidin 3-arabinoside	C21H21O10	Flavonoids	
	17	2.8E-03	1.5	403.109	-	404.111	*[M-H]-*	14	5,3\'-Dihydroxy-3,6,7,4\',5\'-pentamethoxyflavone	C20H20O9	Flavonoids	
	17	5.4E-04	1.5	357.101	-	358.105	*[M-H]-*	8	7-Hydroxy-6,2\',4\',5\'-tetramethoxyisoflavone	C19H18O7	Flavonoids	
	15	1.8E-03	1.7	595.167	-	596.174	*[M-H]-*	0	Naringenin 5,7-di-O-glucoside	C27H32O15	Flavonoids	
	14	4.3E-04	3.4	979.290	-	958.295	*[M+Na-2H]-*	20	Acacetin 7-rhamnosyl-(1->4\'\')[glucosyl-(1->6\'\')(6\'\'\'-acetylsophoroside)]	C42H54O25	Flavonoids	
	277	6.3E-05	2.0	422.087	-	423.095	*[M-H]-*	1	S-(1,2-dicarboxyethyl)glutathione	C14H21N3O10S	Glutathione derivatives	
	23	2.5E-04	2.0	423.091	-	442.116	*[M-H2O-H]-*	16	S-(4-Nitrobenzyl)glutathione	C17H22N4O8S	Glutathione derivatives	
	297	2.0E-05	2.0	194.045	+	193.038	*[M+H]+*	1	5,6-Dihydroxyindole-2-carboxylic acid	C9H7NO4	Indoles	
	115	2.7E-06	2.1	260.056	+	237.064	*[M+Na]+*	11	Methyl 2,3-dihydro-3,5-dihydroxy-2-oxo-3-indoleacetic acid	C11H11NO5	Indoles	
	82	3.7E-04	1.6	150.056	+	149.048	*[M+H]+*	6	3-Hydroxyindolin-2-one	C8H7NO2	Indoles	
	56	8.6E-03	4.6	982.243	+	959.230	*[M+Na]+*	23	(+)-7-Isojasmonic acid CoA	C33H52N7O18P3S	Jasmonates	
	1178	4.0E-04	1.7	493.281	+	470.301	*[M+Na]+*	18	(25R)-26,26,26-trifluoro-1'alpha;,25-dihydroxycholecalciferol	C27H41F3O3	Lipids	Unlikely
	596	4.2E-04	1.7	494.286	+	511.291	*[M+H-H2O]+*	4	Phosphoserine	C23H46NO9P	Lipids	
	162	5.3E-06	2.0	231.048	+	208.059	*[M+Na]+*	1	Lipoic acid	C8H16O2S2	Lipids	
	83	4.8E-04	2.0	824.725	+	859.739	*[M+H-2H2O]+*	1	Phosphatidylcholine	C50H102NO7P	Lipids	
	16	1.5E-04	2.2	165.038	-	166.048	*[M-H]-*	14	Arabinonic acid	C5H10O6	Lipids	
	59	1.2E-07	2.2	165.042	+	142.053	*[M+Na]+*	1	4-Cyanoindole	C9H6N2	Miscellaneous/Drugs	Unlikely
	62	1.6E-04	1.5	438.083	-	439.090	*[M-H]-*	0	Hydroxytinidazole glucuronide	C14H21N3O11S	Miscellaneous/Drugs	Unlikely
	24	2.8E-04	1.5	460.065	-	415.066	*[M+FA-H]-*	0	Cephaloridine	C19H17N3O4S2	Miscellaneous/Drugs	Unlikely
	131	2.7E-05	2.2	275.533	+	527.071	*[M+H+Na]2+*	2	Indoxacarb	C22H17ClF3N3O7	Miscellaneous/Oxazines	Unlikely
	17	8.0E-04	1.5	424.086	-	425.086	*[M-H]-*	16	5,12-Dihydroxanthommatin	C20H15N3O8	Miscellaneous/Oxazines	Unlikely
	15	4.0E-04	3.8	572.058	-	527.071	*[M+FA-H]-*	18	Indoxacarb	C22H17ClF3N3O7	Miscellaneous/Oxazines	Unlikely
	64	5.0E-03	1.6	572.158	+	571.160	*[M+H]+*	16	Cyclochlorotine	C24H31Cl2N5O7	Miscellaneous/Peptides	
	149	5.9E-04	3.8	535.028	+	534.029	*[M+H]+*	15	UDP-L-Ara4O	C14H20N2O16P2	Nucleotides	
	720	1.8E-05	2.0	827.233	+	804.258	*[M+Na]+*	17	5-Formyl-5,6,7,8-tetrahydromethanopterin	C31H45N6O17P	Organic acid	
	381	2.7E-04	2.0	797.222	+	774.247	*[M+Na]+*	18	7,8-Dihydromethanopterin	C30H43N6O16P	Organic acid	
	159	2.8E-04	4.7	538.029	+	515.045	*[M+Na]+*	10	3-(ADP)-glycerate	C13H19N5O13P2	Organic acid derivatives	
	150	4.1E-06	2.2	149.011	-	150.016	*[M-H]-*	15	Tartaric acid	C4H6O6	Organic acids	
	33	1.6E-05	2.1	179.022	-	134.022	*[M+FA-H]-*	14	Malic acid	C4H6O5	Organic acids	
	26	1.8E-04	2.2	150.015	-	169.038	*[M-H2O-H]-*	28	Dihydrodipicolinic acid	C7H7NO4	Organic acids	
	24	4.3E-04	1.5	408.072	-	387.099	*[M+Na-2H]-*	3	Pyraclostrobin	C19H18ClN3O4	Organic acids/Carbamates	Unlikely
	368	4.6E-06	2.1	194.046	-	195.053	*[M-H]-*	1	N-acetyl-4-aminosalicylic acid	C9H9NO4	Phenylpropanoids/SA derivatives	
	189	1.1E-05	1.5	194.045	-	195.053	*[M-H]-*	2	N-acetyl-4-aminosalicylic acid	C9H9NO4	Phenylpropanoids/SA derivatives	
	16	1.5E-04	2.1	195.049	-	214.063	*[M-H2O-H]-*	20	Phenyl salicylate	C13H10O3	Phenylpropanoids/SA derivatives	
	16	4.9E-04	3.8	542.061	-	521.081	*[M+Na-2H]-*	9	4-(Cytidine 5\'-diphospho)-2-C-methyl-D-erythritol	C14H25N3O14P2	Sugar alcohols	
	24	4.2E-03	3.1	671.291	-	650.330	*[M+Na-2H]-*	20	Thapsigargin	C34H50O12	Terpenoids	Unlikely
	7523	1.3E-06	2.2	267.546	+						Unknown	
	518	1.1E-06	2.2	259.557	+						Unknown	
	329	4.5E-06	2.2	268.558	+						Unknown	
	241	5.8E-06	2.2	274.563	+						Unknown	
	168	7.3E-05	2.0	391.588	+						Unknown	
	148	8.8E-04	2.0	607.672	+						Unknown	
	145	1.1E-05	2.2	260.556	+						Unknown	
	106	5.2E-05	2.0	630.666	+						Unknown	
	96	1.8E-05	2.2	282.550	+						Unknown	
	93	2.0E-03	2.0	592.672	+						Unknown	
	87	5.2E-03	2.2	342.578	+						Unknown	
	66	2.3E-05	2.2	350.566	+						Unknown	
	59	8.1E-05	2.0	376.582	+						Unknown	
	58	3.0E-05	2.7	669.273	-						Unknown	
	35	1.0E-04	2.7	670.279	-						Unknown	
	21	1.3E-03	1.5	798.190	-						Unknown	
	17	9.0E-04	2.9	1067.243	-						Unknown	
	15	3.5E-06	2.2	1179.206	-						Unknown	
	14	1.6E-03	1.5	1111.044	-						Unknown	

SOIL-ENRICHED

Sample	FC	*P* value[2]	RT (min)[3]	Detected *m/z*[3]	Ion mode[3]	Predicted mass[4]	*Adduct*[4]	Δppm[4]	Putative Compound[4]	Predicted Formula[4]	Putative Pathway[5]	Soil- or plant-derived[6]
Control soil	0.28	1.8E-03	8.7	309.175	+	286.189	*[M+Na]+*	11	N-Acetyl-leucyl-leucine	C14H26N2O4	Amino acids	
	0.12	1.4E-04	1.2	644.697	-	599.686	*[M+FA-H]-*	20	Calcium trimetaphosphate	Ca3H6O18P6	Calcium ions	
	0.43	6.3E-03	8.6	171.149	+	170.142	*[M+H]+*	1	3-Acrylamidopropyl trimethylammonium	C9H18N2O	Inorganic compounds	Unlikely
	0.04	1.6E-06	6.6	783.518	+	782.510	*[M+H]+*	1	Phosphatidylglycerol	C43H75O10P	Lipids	
	0.19	1.4E-03	6.4	607.394	+	624.400	*[M+H-H2O]+*	5	Phosphatidylglycerol	C31H61O10P	Lipids	
	0.22	4.1E-03	5.5	521.315	+	498.332	*[M+Na]+*	12	Phosphatidylglycerol	C24H51O8P	Lipids	
	0.23	3.2E-03	6.1	588.422	+	565.447	*[M+Na]+*	24	Phosphatidylcholine	C30H64NO6P	Lipids	
	0.24	4.7E-03	6.5	382.315	+	417.324	*[M+H-2H2O]+*	9	N-arachidonoyl leucine	C26H43NO3	Lipids	
	0.25	4.5E-03	5.2	483.292	+	482.285	*[M+H]+*	1	Vitamin D3 derivative (2716)	C30H42O3S	Lipids	Unlikely
	0.26	2.0E-03	6.8	741.523	+	740.536	*[M+H]+*	26	Phosphatidic acid	C42H77O8P	Lipids	
	0.26	2.3E-03	6.6	649.450	+	666.447	*[M+H-H2O]+*	8	Phosphatidylglycerol	C34H67O10P	Lipids	
	0.28	8.3E-03	6.6	693.405	+	670.406	*[M+Na]+*	14	Phosphatidylglycerol	C32H63O12P	Lipids	
	0.29	9.8E-03	6.7	566.452	+	565.447	*[M+H]+*	4	Phosphatidylcholine	C30H64NO6P	Lipids	
	0.30	1.2E-04	8.4	418.271	+	395.282	*[M+Na]+*	1	N-(3-hydroxyphenyl)-Arachidonoyl amide	C26H37NO2	Lipids	
	0.33	9.1E-03	6.9	652.440	+	669.437	*[M+H-H2O]+*	8	Phosphatidylethanolamine	C36H64NO8P	Lipids	
	0.33	7.5E-03	5.3	737.512	+	736.504	*[M+H]+*	0	Phosphatidic acid	C42H73O8P	Lipids	
	0.34	1.8E-04	4.1	405.199	+	382.212	*[M+Na]+*	5	Phosphatidic acid	C17H35O7P	Lipids	
	0.36	7.9E-03	6.0	660.481	+	659.468	*[M+H]+*	9	Diacylglycerol	C43H63D5O5	Lipids	
	0.36	7.0E-03	6.1	629.418	+	628.410	*[M+H]+*	0	Phosphatidic acid	C34H61O8P	Lipids	
	0.37	2.1E-03	8.5	662.465	+	661.459	*[M+H]+*	1	2-carboxy-2-amino-3-O-(13\'-methyltetradecanoyl)-4-hydroxy-17-methyloctadec-5-ene-1-sulfonic acid	C35H67NO8S	Lipids	Unlikely
	0.37	6.4E-03	8.8	1010.743	+	987.759	*[M+Na]+*	4	Galabiosylceramide	C55H105NO13	Lipids	
	0.38	1.1E-03	6.4	648.561	+	647.561	*[M+H]+*	11	Diacylglycerol	C41H75D5O5	Lipids	
	0.38	2.6E-04	8.8	929.775	+	928.752	*[M+H]+*	16	Triacylglycerol	C61H100O6	Lipids	
	0.38	3.1E-03	8.6	518.351	+	495.369	*[M+Na]+*	13	Phosphatidylcholine	C25H54NO6P	Lipids	
	0.40	9.9E-03	5.7	420.283	+	437.291	*[M+H-H2O]+*	11	Phosphatidylethanolamine	C21H44NO6P	Lipids	
	0.40	1.1E-03	8.7	766.578	+	765.567	*[M+H]+*	4	Phosphatidylcholine	C44H80NO7P	Lipids	
	0.41	4.8E-03	7.7	684.483	+	683.474	*[M+H]+*	2	Phosphatidylglycerol	C34H67O10P	Lipids	
	0.41	8.8E-03	6.4	1032.690	+	1009.707	*[M+Na]+*	5	3-O-acetyl-sphingosine-2,3,4,6-tetra-O-acetyl-GalCeramide	C56H99NO14	Lipids	
	0.42	5.8E-03	8.3	764.596	+	799.609	*[M+H-2H2O]+*	0	Phosphatidylethanolamine	C45H86NO8P	Lipids	
	0.44	4.1E-03	6.8	528.394	+	563.395	*[M+H-2H2O]+*	22	Phosphatidylcholine	C29H58NO7P	Lipids	
	0.44	5.7E-03	6.6	902.749	+	901.750	*[M+H]+*	9	Phosphatidylcholine	C52H104NO8P	Lipids	
	0.06	1.4E-04	1.3	691.689	-	692.705	*[M-H]-*	12	Hydroxyphthioceranic acid (C46)	C46H92O3	Lipids	Unlikely
	0.09	9.6E-04	6.3	1154.726	-	1133.780	*[M+Na-2H]-*	24	Galalpha1-4Galbeta1-4Glcbeta-Ceramide	C60H111NO18	Lipids	
	0.20	6.0E-04	7.5	897.675	-	898.705	*[M-H]-*	25	Triacylglycerol	C59H94O6	Lipids	
	0.20	3.4E-03	2.3	719.725	-	720.736	*[M-H]-*	4	Hydroxyphthioceranic acid (C48)	C48H96O3	Lipids	Unlikely
	0.20	4.7E-03	6.8	1007.732	-	1026.768	*[M-H2O-H]-*	17	Triacylglycerol	C69H102O6	Lipids	
	0.22	2.2E-03	8.9	312.836	-	291.860	*[M+Na-2H]-*	4	Ethyl 2-Bromo-2-iodoacetate	C4H6BrIO2	Lipids	Unlikely
	0.23	6.1E-03	8.7	693.481	-	712.504	*[M-H2O-H]-*	6	Phosphatidic acid	C40H73O8P	Lipids	
	0.24	9.1E-03	8.5	647.673	-	648.678	*[M-H]-*	2	Octacosyl-palmitate	C44H88O2	Lipids	
	0.24	1.0E-03	1.2	937.711	-	938.736	*[M-H]-*	19	Triacylglycerol	C62H98O6	Lipids	
	0.42	3.1E-03	7.6	520.315	+	519.294	*[M+H]+*	25	Vignatic acid B	C27H41N3O7	Miscellaneous	Unlikely
	0.44	6.5E-03	6.9	493.453	+	510.455	*[M+H-H2O]+*	1	Nb-Lignoceroyltryptamine	C34H58N2O	Miscellaneous/Alkylindoles	Unlikely
	0.03	3.5E-05	1.1	208.936	-	209.941	*[M-H]-*	13	2,4.6-Trichloroanisole	C7H5Cl3O	Miscellaneous/Aromatics	Unlikely
	0.15	5.5E-04	1.6	596.816	-	597.811	*[M-H]-*	20	Iopodic acid	C12H13I3N2O2	Miscellaneous/Aromatics	Unlikely
	0.22	5.1E-04	1.6	244.894	-	223.920	*[M+Na-2H]-*	3	2,3,6-Trichlorobenzoic acid	C7H3Cl3O2	Miscellaneous/Aromatics	Unlikely
	0.31	8.1E-04	8.6	476.308	+	475.301	*[M+H]+*	0	Netilmicin	C21H41N5O7	Miscellaneous/Drugs	Unlikely
	0.35	2.0E-03	8.6	319.202	+	336.205	*[M+H-H2O]+*	0	Acebutolol	C18H28N2O4	Miscellaneous/Drugs	Unlikely
	0.44	2.7E-03	4.5	450.171	+	427.180	*[M+Na]+*	5	5-hydroxyfluvastatin	C24H26FNO5	Miscellaneous/Drugs	Unlikely
	0.17	4.2E-03	7.7	493.102	-	448.103	*[M+FA-H]-*	1	N,N\'-(((4-methyl-1,3-phenylene)bis(azanediyl))bis(carbonothioyl))dibenzamide	C23H20N4O2S2	Miscellaneous/Drugs	Unlikely
	0.24	4.7E-03	8.8	1015.228	-	994.249	*[M+Na-2H]-*	4	Calcein AM	C46H46N2O23	Miscellaneous/Fluoresceins	Unlikely
	0.00	6.0E-05	0.8	209.949	-	188.975	*[M+Na-2H]-*	2	2,6-Dichlorobenzamide	C7H5Cl2NO	Miscellaneous/Insecticides	Unlikely
	0.01	2.5E-05	1.0	225.926	-	244.950	*[M-H2O-H]-*	21	7-N,N-Dimethylamino-1,2,3,4,5-pentathiocyclooctane	C5H11NS5	Miscellaneous/Insecticides	Unlikely
	0.23	9.5E-03	6.5	633.269	+	668.283	*[M+H-2H2O]+*	2	Filicin	C36H44O12	Miscellaneous/Ketones	Unlikely
	0.29	6.3E-04	6.4	939.584	+	974.593	*[M+H-2H2O]+*	4	Megalomicin C2	C49H86N2O17	Miscellaneous/Macrolides	Unlikely
	0.02	7.0E-03	1.1	306.938	-	325.964	*[M-H2O-H]-*	25	Butonate	C8H14Cl3O5P	Miscellaneous/Organophosphonates	Unlikely
	0.03	3.3E-04	0.9	338.895	-	317.928	*[M+Na-2H]-*	24	Tiludronic acid	C7H9ClO6P2S	Miscellaneous/Organophosphonates	Unlikely
	0.43	7.1E-03	6.9	696.468	+	731.483	*[M+H-2H2O]+*	3	Microcolin B	C39H65N5O8	Miscellaneous/Peptides	Unlikely
	0.21	3.4E-03	9.0	887.232	-	888.232	*[M-H]-*	7	Cyanidin 3-[6-(6-p-coumarylglucosyl)-2-xylosylgalactoside]	C41H44O22	Miscellaneous/Pigments	
	0.29	5.7E-04	8.8	1031.546	+	1066.556	*[M+H-2H2O]+*	2	3-O-(Glcb1-2(Xylb1-3)Glcb1-4Galb)-(25R)-5alpha-spirostan-3beta-ol	C51H86O23	Miscellaneous/Polysaccharides	
	0.44	3.6E-03	8.7	361.237	+	360.230	*[M+H]+*	0	11beta-17-Dihydroxy-6alpha-methylpregn-4-ene-3,20-dione	C22H32O4	Miscellaneous/Steroids	Unlikely
	0.14	2.5E-03	8.9	859.065	-	878.098	*[M-H2O-H]-*	16	Mn(III) tetrakis(4-benzoic acid) porphyrin chloride	C48H28ClMnN4O8	Miscellaneous/Tetrapyrroles	
	0.32	2.4E-03	6.6	500.401	+	535.403	*[M+H-2H2O]+*	22	25-dihydroxycholecalciferol	C35H53NO3	Miscellaneous/Vitamins	Unlikely
	0.40	6.5E-03	6.6	541.358	+	576.366	*[M+H-2H2O]+*	8	1-Hydroxyvitamin D3 3-D-glucopyranoside	C33H52O8	Miscellaneous/Vitamins	Unlikely
	0.16	5.1E-03	5.6	723.070	-	724.065	*[M-H]-*	16	Adenylated molybdopterin	C20H26N10O12P2S2	Nucleotides	
	0.23	4.9E-03	6.7	666.049	-	621.061	*[M+FA-H]-*	14	UDP-N-acetyl-D-galactosaminuronic acid	C17H25N3O18P2	Nucleotides	
	0.21	5.3E-03	1.3	432.844	-	433.851	*[M-H]-*	0	3-(3,5-Diiodo-4-hydroxyphenyl)lactate	C9H8I2O4	Organic acids	
	0.40	1.1E-03	6.4	491.189	+	468.200	*[M+Na]+*	0	Paucin	C23H32O10	Terpenoids	Unlikely
	0.15	1.5E-04	3.9	443.132	-	444.141	*[M-H]-*	3	1-O-[2-(L-Cysteinamido)-2-deoxy-alpha-D-glucopyranosyl]-1D-myo-inositol	C15H28N2O11S	Sugar alcohols	
	0.29	3.3E-03	5.4	383.217	+						Unknown	
	0.30	7.2E-03	1.1	217.987	+						Unknown	
	0.39	4.1E-03	6.4	1049.598	+						Unknown	
	0.40	3.3E-03	6.2	664.622	+						Unknown	
	0.42	9.8E-03	6.5	988.586	+						Unknown	
	0.43	4.7E-04	8.8	768.714	+						Unknown	
	0.45	4.8E-03	6.4	636.900	+						Unknown	
	0.02	9.3E-04	1.1	192.959	-						Unknown	
	0.03	1.8E-03	1.1	322.917	-						Unknown	
	0.06	3.2E-05	1.3	747.725	-						Unknown	
	0.08	1.1E-04	1.3	910.685	-						Unknown	
	0.11	2.9E-03	1.9	629.726	-						Unknown	
	0.14	7.9E-03	2.0	434.817	-						Unknown	
	0.14	1.6E-03	1.9	610.816	-						Unknown	
	0.15	2.1E-03	1.5	319.839	-						Unknown	
	0.17	1.1E-03	1.1	311.877	-						Unknown	
	0.17	2.8E-03	9.1	557.078	-						Unknown	
	0.18	3.8E-04	1.3	328.864	-						Unknown	
	0.18	3.3E-04	1.5	255.929	-						Unknown	
	0.19	1.2E-03	2.1	587.806	-						Unknown	
	0.19	1.7E-03	1.9	568.840	-						Unknown	
	0.19	4.8E-03	1.1	1021.633	-						Unknown	
	0.20	9.8E-03	1.3	782.734	-						Unknown	
	0.20	9.4E-03	1.1	295.900	-						Unknown	
	0.21	3.3E-04	8.9	1098.495	-						Unknown	
	0.21	1.9E-03	2.3	320.835	-						Unknown	
	0.23	3.9E-03	1.3	575.804	-						Unknown	
	0.23	6.0E-04	2.7	652.759	-						Unknown	
	0.24	2.0E-03	1.2	574.803	-						Unknown	
	0.24	1.2E-04	8.9	1178.917	-						Unknown	
	0.24	4.7E-03	9.3	566.779	-						Unknown	
	0.24	7.0E-03	3.9	261.886	-						Unknown	
