# Supplementary material for: Metabolite profiling of non‐sterile rhizosphere soil
Source: Plant J. 2017 Aug 31;92(1):147–62. doi: 10.1111/tpj.13639 (PMC5639361; doi:10.1111/tpj.13639)
Supplement: Supplementary file 12 — Appendix S1. Supplementary experimental procedures. [file TPJ-92-147-s012.docx]

**Appendix S1.** Supplementary experimental procedures.

**DNA extraction, 16S rRNA gene sequencing and analysis.**

For microbial profiling, eight additional growth tubes were set up, as described in the Experimental Procedures, but were not used for the collection of chemicals. Four of these tubes contained one Arabidopsis plant and four contained only growth substrate. After 5 weeks, plants were sampled by carefully loosening the soil around the edges of the growth tube, pulling up the roots and removing excess soil by shaking. Soil samples were also taken from the tubes without plants, using a sterile spatula and avoiding surface material. DNA was extracted from the resulting samples consisting of either roots covered in their closely adhering soil (root plus rhizosphere samples), or only soil (control soil), using a PowerSoil DNA extraction kit (MoBio Laboratories Inc., Carlsbad, CA, USA) according to the manufacturer's instructions. Partial prokaryotic 16S rRNA genes were amplified from this extract, using primers 799F and 1193R (Chelius and Triplett, 2001; Bodenhausen *et al.*, 2013), which were modified to include the Illumina overhang adapter nucleotide sequences (adapters shown in normal typeface, locus specific primers in bold letter font):

799F: TCGTCGGCAGCGTCAGATGTGTATAAGAGACAG**AACMGGATTAGATACCCKG**

1193R:

GTCTCGTGGGCTCGGAGATGTGTATAAGAGACAG**ACGTCATCCCCACCTTCC**.

PCRs were carried out, using 0.4 U of KAPA HiFi HotStart DNA polymerase (**Kapa Biosystems Ltd, London, UK)** on 2 μL of DNA extract in the presence of 2.5 mM MgCl_2_, 1.2 mM deoxynucleoside triphosphates (dNTPs), 0.2 µM of each primer, and the manufacturer's reaction buffer in a total reaction volume of 20 μL (PCR conditions: 95 °C for 3 min; 25 cycles at 95 °C for 30 s, 58 °C for 30 s and 72 °C for 30 s; and 72 °C for 5 min). To reduce PCR bias, the PCR was performed in triplicate and amplicons were pooled. A sequencing library was constructed by cleaning up pooled PCR products, using AMPure XP beads (**Beckman Coulter (UK) Ltd, High Wycomb, UK), followed** by attachment of dual indices and Illumina sequencing adapters, using the Nextera XT Index Kit (Illumina Inc. Essex UK) and following the manufacturer’s instructions. The indexed PCR products were cleaned using AMPure XP beads and sequencing was performed using a paired end 2 x 250 bp cycle kit v2 on a MiSeq machine running v2 chemistry (Illumina Inc, at The Genome Analysis Centre, Norwich, UK). Raw sequencing data were deposited in the European Nucleotide Archive (ENA) under accession number PRJEB17782. Sequences were analysed by USEARCH (Edgar, 2010) and Qiime pipelines (Caporaso *et al.*, 2010a). Sequences were filtered using USEARCH, retaining those with a maxEE value of 1 (equivalent to 1 in 1,000 errors) and 251 bp long. Chimeras were detected using UCHIME (Edgar *et al.*, 2011), using both reference based and *de novo* detection methods. After selection of OTUs by USEARCH (97% similarity), the representative sequences were aligned to the Greengenes 13_8 core reference alignment ([DeSantis *et al.* 2006](#_ENREF_3)) using PyNAST (Caporaso *et al.*, 2010b). All other steps leading to the generation of OTU abundance tables were performed using Qiime. All statistical analyses of community data were performed using the R programming language (R Development Core Team, 2016; <https://www.R-project.org/>) and with the packages phyloseq (McMurdie and Holmes, 2013), vegan (<https://github.com/vegandevs/vegan>) and DESeq2 (Love *et al.*, 2014).

**Quantification of plant tissue damage by electrolytes leakage.**

Tissue damage by the acidified extraction solutions was quantified by conductivity of cell electrolytes leakage, as described previously (Pétriacq *et al.*, 2016a, 2016b). For Arabidopsis, roots were collected from plants cultivated in half strength Murashige-Skoog, solidified with 0.8% Phytagel (Sigma-Aldrich, UK) and adjusted to pH 5.8. Root replicates consisted of one intact root system from 2-week-old plants, which was removed carefully from the agar medium. For maize, roots were collected from surface sterilised seeds, germinated and grown for five days on wetted filter paper in sealed petri-dishes. Tissues were incubated for 1 min in 10 mL of different acidified extraction solutions, containing 0.05% formic acid (v/v) and 0%, 50% or 95% methanol (v/v). As a negative control, tissues were incubated in double-distilled sterile water. As a positive control for cell damage, tissues were wounded prior to extraction solution incubation by cutting roots into 10 pieces with a razor blade. Directly after incubation, tissues were rinsed in double-distilled sterile water, then transferred into glass bottles containing 5 mL of double-distilled sterile water, and subsequently agitated at room temperature for 2 hours on an orbital shaker (200 rpm). Conductivity was then measured in the balanced solution, using a CMD 500 WPA conductivity meter. Subsequently, all samples were boiled for 30 min and re-measured for conductivity of lysed tissue. Cell damage was expressed as the average level of conductivity, relative to the maximum level of conductivity after tissue lysis (set at 100%). Each treatment was based on 4 replicated samples (*n* = 4). Data were analysed in IBM SPSS (v. 22), using a Welch’s F test for ranked data, followed by Games-Howell tests to assess individual differences (*P* < 0.05). The experiments were repeated three times with similar results.

**Analysis of microscopic root cell damage by extraction solutions.**

Transgenic Arabidopsis plants (Col-0) expressing the *35S:IBI1:YFP* construct, encoding the cytoplasmic aspartyl-tRNA synthatase IBI1 with a C-terminal fusion to Yellow Fluorescent Protein (Luna *et al.*, 2014; *35S::IBI1:YFP*), were cultivated for two weeks (8.5/15.5 h light/dark at 21/19 °C, 120 µmol m^-2^ s^-1^ photons, 70% relative humidity) on half strength Murashige-Skoog agar plates, solidified with 0.8% Phytagel (Sigma-Aldrich, UK) and adjusted to pH 5.8. Roots were extracted carefully form agar plates, and incubated for 1 min in the acidified MeOH-containing extraction solutions (0, 50, 95% MeOH with 0.05% formic acid, v/v). As negative and positive controls for cell damage, roots were incubated for 1 min in double-distilled sterile water, or for 15 min in 100% MeOH, respectively. After incubation, roots were rinsed in double-distilled sterile water prior to epi-fluorescence microscopy analysis. Fluorescence was observed using an epi-fluorescence microscope (Olympus BX51, excitation filter BP 470/40 nm, barrier filter BP 525/50 nm). For each treatment, root systems form 12 different plants were analysed and photos were taken of representative samples. The experiment was performed four times with similar results.

**Analysis of impacts on soil and rhizosphere bacteria by extraction solutions.**

Tubes (30 mL; *n* = 3) containing the sand:compost mixture (9:1 v/v) with or without 5-week-old Arabidopsis were left untreated, or were bacterized by syringe injection with 5 mL of 10 mM MgSO_4_, containing either YFP-expressing *P. simiae* WCS417r (Zamioudis *et al.*, 2014), or rifampicin-resistant *B. subtilis* 168 (Yi *et al.*, 2016), to a final density of 10^7^ colony CFU g^-1^. After 48 h, tubes were flushed with extraction solution (as detailed in Experimental procedures). Additional tubes were flushed with double-distilled sterile water (control), or 95% MeOH and left for 45 min (positive control for cell damage). Subsequently, 1 g of either control soil (without roots), or Arabidopsis roots plus adhering rhizosphere soil, was sampled from the tubes, suspended for 5 min into 50 mL of 10 mM MgSO_4_, and centrifuged (5 min, 3,500 *g*). Pellets were re-suspended in 1 mL of 10 mM MgSO_4_, and subjected to dilution plating onto Luria Broth (LB) agar medium supplemented with 5 µg mL^-1^ of the anti-fungal cycloheximide. For testing impacts on culturable soil bacteria, LB agar contained no further antibiotics; for testing impacts on *P. simiae* WCS417r and *B. subtilis* 168, plates were supplemented with 5 µg mL^-1^ tetracycline + 25 µg mL^-1^ rifampicin and 50 µg mL^-1^ rifampicin, respectively. Plates were kept for 24 - 48 h at 28 °C. Each biologically replicated sample was plated four times, after which the technical replicates were averaged to minimize confounding effects of heterogeneity in suspended pellets. Experiments were repeated twice with comparable results.

**UPLC-Q-TOF mass spectrometry.**

Untargeted metabolic profiling by UPLC-Q-TOF mass spectrometry (MS) was performed as described previously (Pétriacq *et al.*, 2016b) using an ACQUITY ultra-high-pressure liquid chromatography (UPLC) system coupled to a SYNAPT G2 Q-TOF mass spectrometer with an electrospray (ESI) ionization source (Waters, UK). The system was controlled by MassLynx v. 4.1 software (Waters). Chromatographic separation of samples was carried out at a flow rate of 0.4 mL min^−1^ using an ACQUITY UPLC BEH C18 column (2.1 × 50 mm, 1.7 μm, Waters) coupled to a C18 VanGuard pre-column (2.1 x 5 mm, 1.7 µm, Waters). The mobile phase consisted of solvent A (0.05 %, formic acid v/v, in water) and solvent B (0.05 % formic acid v/v in acetonitrile) with the following gradient: 0 – 3 min 5 – 35 % B, 3 – 6 min 35 – 100 % B, holding at 100 % B for 2 min, 8 – 10 min, 100 – 5 % B. The column was maintained at 45 °C and the injection volume was 10 μL. Between each condition, a blank was injected with 50% methanol (v/v) to clean the column. Sample runs in negative and positive ionization mode (ESI^-^ and ESI^+^) were separated by two consecutive injections with 50% methanol (v/v) to allow stabilization of the ionization modes. An ACQUITY PDA detector (Waters) was used to monitor the UV trace (range 205 – 400 nm, sampling rate 40 points s^-1^, resolution 1.2 nm). MS detection of ions was operated in sensitivity mode by SYNAPT G2 (50 - 1200 Da, scan time = 0.2 s) in both ESI^-^ and ESI^+^, using a full MS scan (*i.e.* no collision energy) and applying the MS^E^ function with a ramp in the transfer cell in elevated energy mode (5 to 45 eV). The following conditions were applied for ESI^-^ (capillary voltage - 3 kV, sampling cone voltage - 25 V, extraction cone voltage -4.5 V, source temperature 120 °C, desolvation temperature 350 °C, desolvation gas flow 800 L h^-1^, cone gas flow 60 L h^-1^), and for ESI^+^ (capillary voltage 3.5 kV, sampling cone voltage 25 V, extraction cone voltage 4.5 V, source temperature 120 °C, desolvation temperature 350 °C, desolvation gas flow 800 L h^-1^, cone gas flow 60 L h^-1^). Prior to analyses, the Q-TOF was calibrated by infusing a sodium formate solution. Accurate mass detection was ensured by infusing the internal lockmass reference peptide leucine enkephalin during each run.

**Statistical analysis of MS data.**

Prior to multivariate analyses, the XCMS R package (v. 3.1.3; Smith *et al.*, 2006) was used to align and integrate raw UPLC-Q-TOF peaks, to correct for total ion current (TIC) and median fold-change. All statistical analyses were performed with median-normalized, cube-root-transformed and Pareto-scaled data, using MetaboAnalyst software (v. 3.0, [http://www.metaboanalyst.ca](http://www.metaboanalyst.ca/); Xia *et al.*, 2015). Three-dimensional principal component analyses (3D-PCA) were based on the first three principal components (PCs) that explain most variation of the dataset. Supervised partial least square discriminant analyses (PLS-DAs) were conducted to quantify discriminative power between soil types and extraction solutions. PLS-DA models were validated by correlation (R^2^) and predictability (Q^2^) parameters for both ESI^+^ and ESI^-^ modes (R^2^ > 0.94 and Q^2^ > 0.59, respectively). Numbers of total ions were obtained from XCMS output datasets. To quantify metabolic differences between rhizosphere and control soil, volcano plots were constructed at a statistically significant threshold of *P* < 0.01 (Welch’s *t*-test) and a fold-difference threshold of 2, using MetaboAnalyst (v. 3.0, [http://www.metaboanalyst.ca](http://www.metaboanalyst.ca/); Xia *et al.*, 2015). To obtain putative identities of a combined set of ions from all three extraction solutions that are either enriched in Arabidopsis soil, or its corresponding control soil, the top-20 ranking ions from each volcano plot were selected by fold-change (above 2 or below -2) and *P* value, followed by an ANOVA (*P* < 0.01) for statistical differences between all soil/extraction solution combinations, using a Benjamini-Hochberg false discovery rate (FDR) correction for multiple hypothesis testing (Hochberg and Benjamini, 1990). To obtain putative identities from the 50% MeOH extraction solution that are either enriched in the maize rhizosphere, or corresponding control soil, the top-50 ranking ions from each volcano plot (ESI^+^ and ESI^-^) were selected. For both cultivation systems, ions were corrected for adducts and/or isotopes, using MarVis (v. 1.0; [http://marvis.gobics.de](http://marvis.gobics.de/); tolerance: *m*/z = 0.1 Da, RT = 10 s; Kaever *et al.*, 2012). Putative metabolites were identified by referencing the final set of detected accurate *m/z* values against publicly available chemical databases using METLIN, PubChem, MassBank, Lipid Bank, ChemSpider, Kegg, AraCyc and MetaCyc database, as documented in several studies (Kaever *et al.*, 2009; Kaever *et al.*, 2012; Gamir *et al.*, 2014a, 2014b; Pastor *et al.*, 2014; Pétriacq *et al.*, 2016a, 2016b). METLIN ([https://metlin.scripps.edu](https://metlin.scripps.edu/)) was used to determine accuracy and chemical formulae for the putative compounds. PubChem (<https://pubchem.ncbi.nlm.nih.gov/>) was used to check the predicted pathway classification. In cases where multiple ions could be annotated to the same putative metabolite (due to different adducts and ionization modes; Tables S1 and S2), they were counted additively to the metabolite class presented in the pie-charts of Figures 6 and 7.

**Experimental system for profiling distant rhizosphere fractions.**

To investigate whether the chemical influence of the rhizosphere extends beyond soil that is closely associated with roots, maize plants were grown in mesh bags, which allowed for physical separation of root systems from the distal soil in the periphery of the growth tube. Bags were constructed from a nylon mesh (35 $\mu$m diameter holes), folded over and heat sealed to produce bags (6 cm x 11 cm, approximate diameter when filled = 3.5 cm). These bags were filled with 85 cm^3^ of a mixture of 75:25 (v/v) agricultural soil:perlite, as used previously for maize experiments. Each mesh bag was placed into the centre of the 150-mL plastic tube (11 cm high and 5 cm diameter; Starlab) with a miracloth sheet covering the bottom hole of the tube. Seventy cm^3^ of the same soil substrate was used to fill the peripheral space between the mesh bag and tube wall. A total of 24 pots were set up in this manner. Pre-germinated maize seeds (W22) were planted into the bags of 12 tubes. The other 12 tubes were left unplanted to serve as plant-free controls. All tubes were wrapped in foil and covered with black plastic beads to prevent algal growth. Sixty mL of distilled water was added to each tube to saturate the soil with water before seeds were planted, after which all pots were transferred to a growth cabinet with the following conditions: 16/8 h light/dark with an average light intensity of 140 µmol m^-2^ s^-1^ at the top of the collection tubes, a relative humidity of 60%, and a constant temperature of 20 °C. Soil metabolites were extracted from the different soil fractions after 24 days of growth. To collect metabolites from the distal soil fractions, black beads were removed, and mesh bags were carefully removed from half of the pots (6 tubes with maize and 6 without). The remaining distal soil in the tube (*i.e.* the soil that had been outside the bag) was tapped to the bottom of the 150-mL tubes and extracted by applying 25 mL of acidified 50% (v/v) MeOH to the top of the soil. The solution was flushed through the tube by applying pressure for 1 min through a modified 150-mL tube lid containing a 50-mL syringe, until ~10 mL of solution was collected from the base of the tube into new 50-mL tubes. To collect metabolites from the whole soil fractions, plastic beads were removed from the remaining 12 pots and maize shoots were cut from the 6 that contained plants. Subsequently, 50 mL of acidified 50% MeOH (v/v) was applied to the top of the tube, keeping the mesh bags in place. The solution was flushed through by applying pressure for 1 min using the modified lid, as previously described, resulting in a least 10 mL of collection volume at the base of the tube. All extracts were centrifuged to pellet soil residues (5 min, 3,500 *g*), after which 8 mL of supernatant were transferred into a new 15-mL centrifuge tube and flash-frozen in liquid nitrogen. All samples were freeze-dried for two days, after which dried material was re-suspended in 500 µL of methanol: water: formic acid (50: 49.9: 0.1, v/v/v), sonicated at 4 °C for 20 min, vortexed, transferred into 2-mL microtubes and centrifuged (15 min, 14,000 *g*, 4 °C). Final supernatants (180 µL) were transferred into glass vials containing a glass insert before injection through the UPLC system. UPLC-Q-TOF analysis was conducted in ESI^-^ as described above. For DIMBOA targeted quantitation, a purified and NMR-verified standard (Ahmad *et al.*, 2011) was run alongside the samples. Metabolomics data were normalised for soil amount (*n* = 6), and subsequent analysis performed with MetaboAnalyst (v. 3.0), as described above (*i.e.* median normalisation, cube-root transformation, Pareto scaling).

**References for the supplementary experimental procedures.**

**Ahmad, S., Veyrat, N., Gordon-Weeks, R., et al.** (2011) Benzoxazinoid metabolites regulate innate immunity against aphids and fungi in maize. *Plant Physiol.*, **157**, 317–327.

**Bodenhausen, N., Horton, M.W. and Bergelson, J.** (2013) Bacterial communities associated with the leaves and the roots of Arabidopsis thaliana. *PloS One*, **8**, e56329.

**Caporaso, J.G., Kuczynski, J., Stombaugh, J., et al.** (2010a) QIIME allows analysis of high-throughput community sequencing data. *Nat. Methods*, **7**, 335–336.

**Caporaso, J.G., Bittinger, K., Bushman, F.D., DeSantis, T.Z., Andersen, G.L. and Knight, R.** (2010b) PyNAST: a flexible tool for aligning sequences to a template alignment. *Bioinforma. Oxf. Engl.*, **26**, 266–267.

**Chelius, M.K. and Triplett, E.W.** (2001) The Diversity of Archaea and Bacteria in Association with the Roots of Zea mays L. *Microb. Ecol.*, **41**, 252–263.

**Edgar, R.C.** (2010) Search and clustering orders of magnitude faster than BLAST. *Bioinforma. Oxf. Engl.*, **26**, 2460–2461.

**Edgar, R.C., Haas, B.J., Clemente, J.C., Quince, C. and Knight, R.** (2011) UCHIME improves sensitivity and speed of chimera detection. *Bioinforma. Oxf. Engl.*, **27**, 2194–2200.

**Gamir, J., Pastor, V., Kaever, A., Cerezo, M. and Flors, V.** (2014) Targeting novel chemical and constitutive primed metabolites against Plectosphaerella cucumerina. *Plant J.*, **78**, 227–240.

**Hochberg, Y. and Benjamini, Y.** (1990) More powerful procedures for multiple significance testing. *Stat. Med.*, **9**, 811–818.

**Kaever, A., Lingner, T., Feussner, K., Göbel, C., Feussner, I. and Meinicke, P.** (2009) MarVis: a tool for clustering and visualization of metabolic biomarkers. *BMC Bioinformatics*, **10**, 92.

**Kaever, A., Landesfeind, M., Possienke, M., Feussner, K., Feussner, I. and Meinicke, P.** (2012) MarVis-Filter: ranking, filtering, adduct and isotope correction of mass spectrometry data. *J. Biomed. Biotechnol.*, **2012**, 263910.

**Love, M.I., Huber, W. and Anders, S.** (2014) Moderated estimation of fold change and dispersion for RNA-seq data with DESeq2. *Genome Biol.*, **15**, 550.

**Luna, E., Hulten, M. van, Zhang, Y., et al.** (2014) Plant perception of β-aminobutyric acid is mediated by an aspartyl-tRNA synthetase. *Nat. Chem. Biol.*, **10**, 450–6.

**McMurdie, P.J. and Holmes, S.** (2013) phyloseq: An R Package for Reproducible Interactive Analysis and Graphics of Microbiome Census Data M. Watson, ed. *PLoS ONE*, **8**, e61217.

**Pastor, V., Gamir, J., Camañes, G., Cerezo, M., Sánchez-Bel, P. and Flors, V.** (2014) Disruption of the ammonium transporter AMT1.1 alters basal defenses generating resistance against Pseudomonas syringae and Plectosphaerella cucumerina. *Front. Plant Sci.*, **5**, 231.

**Pétriacq, P., Stassen, J.H. and Ton, J.** (2016a) Spore density determines infection strategy by the plant-pathogenic fungus Plectosphaerella cucumerina. *Plant Physiol.*, **170**, 2325–2339.

**Pétriacq, P., Ton, J., Patrit, O., Tcherkez, G. and Gakière, B.** (2016b) NAD acts as an integral regulator of multiple defense layers. *Plant Physiol.*, **172**, 1465-1479.

**Smith, C.A., Want, E.J., O’Maille, G., Abagyan, R. and Siuzdak, G.** (2006) XCMS: processing mass spectrometry data for metabolite profiling using nonlinear peak alignment, matching, and identification. *Anal. Chem.*, **78**, 779–787.

**Xia, J., Sinelnikov, I.V., Han, B. and Wishart, D.S.** (2015) MetaboAnalyst 3.0—making metabolomics more meaningful. *Nucleic Acids Res.*, **43**, W251–W257.

**Yi, H.-S., Ahn, Y.-R., Song, G.C., Ghim, S.-Y., Lee, S., Lee, G. and Ryu, C.-M.** (2016) Impact of a Bacterial Volatile 2,3-Butanediol on Bacillus subtilis Rhizosphere Robustness. *Front. Microbiol.*, **7**, 993.

**Zamioudis, C., Hanson, J. and Pieterse, M.J.** (2014) b -Glucosidase BGLU42 is a MYB72-dependent key regulator of rhizobacteria-induced systemic resistance and modulates iron deficiency responses in Arabidopsis roots. *New Phytol.*, **204**, 368–379.
