# Supplementary material for: Metabolite profiling of non‐sterile rhizosphere soil
Source: Plant J. 2017 Aug 31;92(1):147–62. doi: 10.1111/tpj.13639 (PMC5639361; doi:10.1111/tpj.13639)
Supplement: Supplementary file 13 [file TPJ-92-147-s013.docx]

**SUPPORTING INFORMATION**

**Supplemental Figure S1.** Rarefaction curves of detected OTUs.

Shown are curves after removal of singletons for replicate root + rhizosphere samples (green) and control soil samples (red).

**Supplemental Figure S2.** Relative abundance (%) of selected families in control soil samples (‘Soil’; red) and root + rhizosphere samples (‘Root’; green) from the Arabidopsis growth system.

Shown are families containing OTUs with relative abundances > 2% in one or more samples. Each bar represents an individual biological replicate. NA, taxonomy not available.

**Supplemental Figure S3.** Model of expected impacts of solvent polarity on the extraction of soil metabolites.

(**a**) Examples of solvent polarities and their impact on the type of metabolites extracted. Polarity index of water, methanol (MeOH) and hexane are shown within parentheses.

(**b**) Hypothesized impact of solvent polarity on cell damage of plant roots and soil microbes.

**Supplemental Figure S4.** Epi-fluorescence microscopy analysis of cell damage in Arabidopsis roots after exposure to MeOH-containing extraction solutions.

Transgenic roots producing the cytoplasmic aspartyl-tRNA synthase IBI1 fused to YFP (*35S::IBI1:YFP*; Luna *et al.*, 2014) were incubated for 1 min in water or acidified extraction solutions with increasing MeOH concentration (0, 50 or 95% MeOH, v/v + 0.05% formic acid, v/v). After incubation, roots were then rinsed in sterile water, and analysed for YFP fluorescence. Photographs show representative examples from observations of at least 12 roots for each treatment. As a positive control for cell damage, roots were incubated in 100% MeOH for 15 min. The experiment was performed four time with similar results. Scale bars: 50 µm.

**Supplemental Figure S5.** Reproducibility of differences in metabolite profiles between control and Arabidopsis soil over three independent experiments.

Shown are unsupervised three-dimensional principal component analyses (3D-PCA) from extracts by the different solutions (indicated by % MeOH). Ions (*m/z* values) were obtained by UPLC-Q-TOF in positive (ESI^+^, left panels) and negative (ESI^-^, right panel) ionization modes. Analysis was carried out with MetaboAnalyst (v. 3.0), after median normalization, cube-root transformation and Pareto scaling of data. In parentheses are shown the percentages of variation explained by each principal component.

**Supplemental Figure S6**. Binary PLS-DA analysis of metabolite profiles from control soil and Arabidopsis soil for different extraction solutions (indicated by % MeOH).

Ions (*m/z* values) were obtained by UPLC-Q-TOF analysis in both positive (ESI^+^, left panels) and negative (ESI^-^, right panel) ionization mode. Prior to analysis, data were median-normalized, cube-root-transformed and Pareto-scaled. All R^2^ (correlation) and Q^2^ (predictability) values of PLS-DA models were above 0.94 and 0.59, respectively.

**Supplemental Figure S7.** Quantitative differences in detected ions (UPLC-Q-TOF) between extracts from control and Arabidopsis soil.

(**a**) Total numbers of ions (top) detected in Arabidopsis soil and control soil after extraction with the different extraction solutions (indicated by % MeOH). Venn diagrams (bottom) show overlap in total ion numbers between extracts for each extraction solution.

(**b**) Venn diagrams showing overlap in cations (ESI^+^) and anions (ESI^-^) that are statistically different between control and Arabidopsis soil (left panel; *P* < 0.01, Welch’s *t*-test), that are enriched in extracts from Arabidopsis soil (middle panel; > 2-fold enrichment to soil at *P* < 0.01, Welch’s *t*-test), and that enriched are in extracts from control soil (right panel; < 2-fold enrichment to soil at *P* < 0.01, Welch’s *t*-test).

**Supplemental Figure S8.** Relative quantities of selected benzoxazinoid ions in extracts from maize soil and corresponding control soil.

Selective ions (*m/z*) of HBOA (2-hydroxy-4H-1,4-benzoxazin-3-one), DIBOA (2,4-dihydroxy-1,4-benzoxazin-3-one) and 2-hydroxy-7-methoxy-2H-1,4-benzoxazin-3(4H)-one were detected on the basis of retention time and *m/z* value, using UPLC-Q-TOF (ESI^+^, Δppm = 0). Charts indicate means of relative abundances (*n* = 5, ± SEM). Levels of statistical significance are indicated in red above the corresponding bars (Student’s *t*-test).

**Supplemental Figure S9.** Profiling distal rhizosphere chemistry.

(**a**) Experimental growth system to profile chemistry of distal rhizosphere fractions. Maize was grown within nylon mesh bags inside 150-mL tubes, containing agricultural soil from arable farmland and perlite (75:25, v/v). Similar plant-free tubes were constructed as controls. After 24 days of growth, chemicals were extracted with the 50% MeOH solution from either the entire pot (whole soil), or the soil surrounding the root containing mesh bag after its careful removal (distal soil).

(**b**) Binary PCAs showing chemical rhizosphere effects in whole soil fractions (upper panel; short + long distance influence) and distal soil fractions (lower panel; long distance influence), illustrating that the rhizosphere extends beyond soil that is closely associated with roots.

(**c**) Targeted quantification of DIMBOA by UPLC-Q-TOF. Shown are average ion intensities (± SEM; *n* = 6), normalised by soil weight. Letters indicate statistically significant differences between soil types (Student’s *t*-test, *P* < 0.05).

**Supplemental Table S1.** Putative identities of ions enriched in Arabidopsis soil and corresponding control soil.

^1^ Percentages indicate relative MeOH contents of the acidified extraction solutions.

^2^ *P* values are derived from ANOVA followed by false discovery rate correction (Benjamini-Hochberg).

^3^ Retention times (RT) and accurate *m/z* values, detected by UPLC-Q-TOF in negative (-) or positive (+) ion mode.

^4^ Predicted parameters were derived from the METLIN database, using accurate *m/z* values.

^5^ Putative metabolites and their corresponding pathways were validated by information from the PubMed chemical database.

^6^ Putative metabolites that unlikely accumulate in (rhizosphere) soil.

|  |  |  |  |  |  |  |  |  |  |  |  |  |  |  |  |  |  |  |  |
| --- | --- | --- | --- | --- | --- | --- | --- | --- | --- | --- | --- | --- | --- | --- | --- | --- | --- | --- | --- |

**Supplemental Table S2.** Putative identities of ions enriched in maize soil and corresponding control soil.

^1^ Fold-change between maize rhizosphere samples and control soil samples.

^2^ *P* values are derived from Welch’s *t*-test.

^3^ Retention times (RT) and accurate *m/z* values, detected by UPLC-Q-TOF in negative (-) or positive (+) ion mode.

^4^ Predicted parameters were derived from the METLIN database, using accurate *m/z* values.

^5^ Putative metabolites and their corresponding pathways were validated by information from the PubMed chemical database.

^6^ Putative metabolites that unlikely accumulate in (rhizosphere) soil.
